# Antimicrobial Triterpenoids and Ingol Diterpenes from Propolis of Semi-Arid Region of Morocco

**DOI:** 10.3390/molecules27072206

**Published:** 2022-03-28

**Authors:** Ralitsa Chimshirova, Milena Popova, Amina Chakir, Violeta Valcheva, Simeon Dimitrov, Boryana Trusheva, Abderrahmane Romane, Vassya Bankova

**Affiliations:** 1 Institute of Organic Chemistry with Centre of Phytochemistry, Bulgarian Academy of Sciences, Acad. G. Bonchev Str., Bl. 9, 1113 Sofia, Bulgaria; ralitsa.chimshirova@orgchm.bas.bg (R.C.); boryana.trusheva@orgchm.bas.bg (B.T.); vassya.bankova@orgchm.bas.bg (V.B.); 2 Laboratory of Applied Chemistry, Faculty of Sciences Semlalia, Cadi Ayyad University, Boulevard Prince My Abdellah B.P. 2390, Marrakech 40000, Morocco; chakir.amina76@gmail.com (A.C.); romane@uca.ac.ma (A.R.); 3 Department of Infectious Microbiology, The Stephan Angeloff Institute of Microbiology, Bulgarian Academy of Sciences, Acad. G. Bonchev St., Bl. 26, 1113 Sofia, Bulgaria; violeta_valcheva@mail.bg (V.V.); s_dimitrov@microbio.bas.bg (S.D.)

**Keywords:** propolis, triterpenoids, ingol diterpenes, antimicrobial activity, *Euphorbia* spp., Morocco

## Abstract

The chemical composition and antimicrobial activity of propolis from a semi-arid region of Morocco were investigated. Fifteen compounds, including triterpenoids (**1**, **2**, **7**–**12**), macrocyclic diterpenes of ingol type (**3**–**6**) and aromatic derivatives (**13**–**15**), were isolated by various chromatographic methods. Their structures were elucidated by a combination of spectroscopic and chiroptical methods. Compounds **1** and **3** are new natural compounds, and **2**, **4**–**6**, and **9**–**11** are newly isolated from propolis. Moreover, the full nuclear magnetic resonance (NMR) assignments of three of the known compounds (**2**, **4** and **5**) were reported for the first time. Most of the compounds tested, especially the diterpenes **3**, **4**, and **6**, exhibited very good activity against different strains of bacteria and fungi. Compound **3** showed the strongest activity with minimum inhibitory concentrations (MICs) in the range of 4–64 µg/mL. The combination of isolated triterpenoids and ingol diterpenes was found to be characteristic for *Euphorbia* spp., and *Euphorbia officinarum* subsp. *echinus* could be suggested as a probable and new plant source of propolis.

## 1. Introduction

Plants and plant-derived products have a long history of use as therapeutic agents and sources of drug leads. Propolis (bee glue) is considered a plant-derived product since its main and biologically active ingredients are plant secretions. Bees collect resins and exudates from different parts of the plants, and after bringing them to the hive, they mix them with beeswax [1]. The resulting product called propolis is used by the bees as a protective barrier of the hive against pathogenic microorganisms and was recently shown as an essential element of the bee colony’s social immunity [2]. Humans have recognized propolis as a healing substance since ancient times, and nowadays, it is still one of the most frequently used natural remedies [3,4]. Propolis has also found a place as an active ingredient in various cosmetic, food and pharmaceutical preparations [5,6,7], widely available on the market.

The broad application of propolis is due to its multiple biological activities, such as antimicrobial, antioxidant, antitumor, antiviral, anti-inflammatory and immunomodulatory [8,9], among others. Since propolis has been shown to possess both antiviral and anti-inflammatory activity, its perspective as a complementary treatment in patients with SARS-CoV-2 was also studied and underlined [10,11,12].

The beneficial effects of propolis are attributed to various plant metabolites as its chemistry depends strongly on the vegetation around the beehives [13]. In fact, the biodiversity of the flora in different geographical and climatic regions reflects in very complex and diverse propolis chemistry, resulting in isolation and characterization of a number of compounds, including numerous new molecules. Thus, propolis appears as a valuable natural product that provides access to plant metabolites, which would be otherwise difficult to discover among the plant biodiversity and/or without destroying the plants.

On the other hand, since propolis is one of the commercial bee products, the knowledge of the chemical composition of propolis from different regions is of primary importance with respect to its pharmacological efficacy and safety use [14]. The chemical variability and the need for standardization and quality control lead to research efforts on the classification of propolis into specific types based on its chemical composition and botanical source(s). Until now, several propolis types have been formulated, among which are those coming from *Populus* spp., mainly *Populus nigra* L. in temperate regions [13,15,16], *Baccharis dracunculifolia* DC. [17,18] and *Dalbergia ecastaphyllum* (L.) Taub [19,20] in Brazil, *Mangifera indica* L. in different subtropical and tropical regions [21,22] and *Cupressus sempervirens* L. in The Mediterranean [23]. Chemical constituents such as flavonoids, prenylated *p*-coumaric acids, isoflavonoids, phenolic lipids and labdane diterpenes, respectively, are amongst their main active principles.

Morocco is a country with Mediterranean and Sub-mediterranean climate on the North of Atlas mountain range and Semi-arid climate in the South. Phytochemical studies showed that samples collected from northern and central regions of the country belong to the poplar and Mediterranean propolis types [24,25]. To the best of our knowledge, however, the chemistry of propolis of the semi-arid regions of Morocco has never been reported. Due to the hot desert climate, the flora of these regions is represented mainly by shrubs and cactiforms, and beekeeping is an important agricultural activity [26,27,28].

The aim of the current study was to conduct a detailed chemical analysis of propolis originating from a semi-arid region of Morocco and reveal its antimicrobial activity and botanical source. As a result, we reported on the isolation and characterization of antimicrobial triterpenoids and ingol type diterpenes, a combination typical for the latex of *Euphorbia* cactiforms.

## 2. Results 

### 2.1. Structural Elucidation of Isolated Compounds

**Figure 1:**
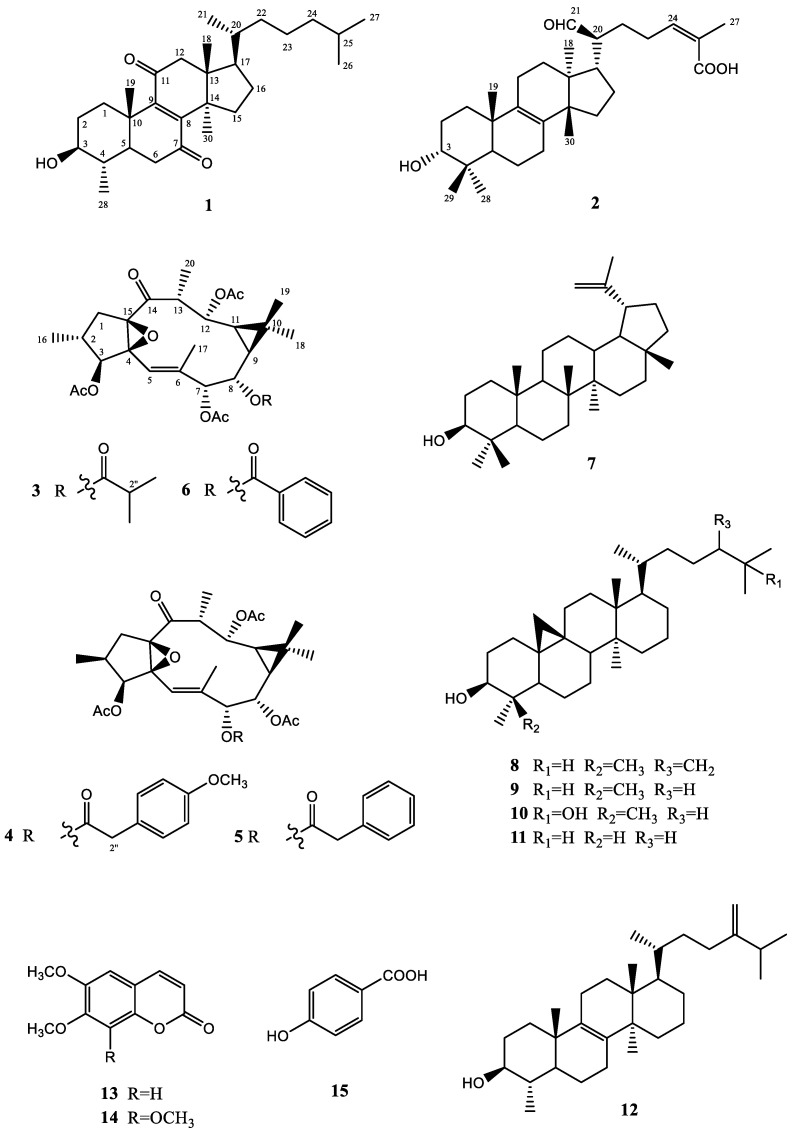
Structures of the isolated compounds.

Detailed chemical analysis of Moroccan propolis sample collected in Sidi-Ifni province was performed. By using different chromatographic procedures, a total of 15 constituents (Figure 1) were isolated: triterpenoids, macrocyclic diterpenes and aromatic derivatives. Their structures were elucidated by a combination of one-dimensional (1D) and two-dimensional (2D) nuclear magnetic resonance (NMR) spectroscopy, high-resolution electrospray ionization mass spectrometry (HRESIMS), optical rotation and comparison with the literature data.

**Figure 2:**
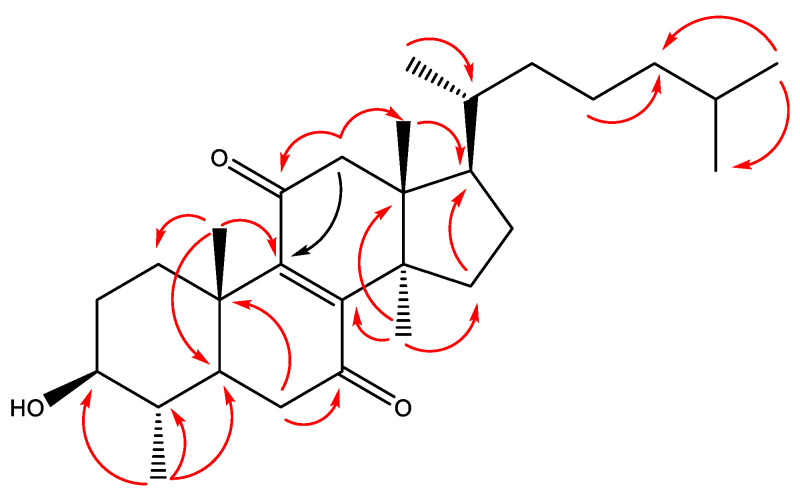
Key HMBC (H→C) correlations of compound **1**.

Compound **1** was isolated as a white amorphous powder. Its HRESIMS displayed a protonated molecule [M + H]^+^ at *m*/*z* 443.3527 (Figure S1, Supplementary Materails), corresponding to molecular formula C_29_H_46_O_3_. The ^1^H and ^13^C NMR spectra showed signals in the range of *δ*_H_ 0.80–3.20 (including signals for 7 CH_3_ groups) and signals for a total of 29 carbon atoms, respectively, suggesting that **1** is a nortriterpenoid (Table 1). Moreover, the signal at *δ*_H_ 3.13, observed as *ddd* with *J* 11.2, 9.7 and 5.1 Hz, was an indication for an axial proton at C-3 in 3*β*-hydroxy-29-nortriterpene [29]. This was supported by the HMBC correlations of methyl protons at *δ*_H_ 1.01 (d, *J* = 6.3 Hz, CH_3_-28) to one oxygenated sp^3^ carbon at *δ*_c_ 75.4 (C-3) and two other sp^3^ carbons at *δ*_c_ 38.2 (C-4) and 47.1 (C-5) (Figure 2). In addition, the NMR data showed the presence of *α*,*β*-unsaturated diketone fragment: signals for two downshifted CH_2_ groups at *δ*_H_ 2.51 (dd, *J* = 15.4 and 2.7 Hz, 1H, H-6a)/*δ*_H_ 2.29 (t, *J* = 15.1 Hz, 1H, H-6b) and *δ*_H_ 2.76 (dd, *J* = 16.0 and 0.9 Hz, 1H, H-12a)/*δ*_H_ 2.64 (brd, *J* = 16.0 Hz, 1H, H-12b), protons of which showed HMBC correlations to carbons at *δ*_c_ 201.6 (C-7)/151.3 (C-8) and 202.8 (C-11)/151.5 (C-9), respectively. The remaining signals in the 1D NMR spectra, in combination with HSQC data, corresponding to a total of six methyl (three of them doublets), seven methylene and three methine groups, and three quaternary sp^3^ carbons, which is in accordance with tetracyclic triterpene skeleton with saturated (C_8_H_17_) side chain. The position of the *α*,*β*-unsaturated diketone fragment was further confirmed by the HMBC cross-peaks of H_3_-19/C-9, H_2_-12/C-9 and C-11, and H_3_-30/C-8 and H_2_-6/C-7.

**Figure 3:**
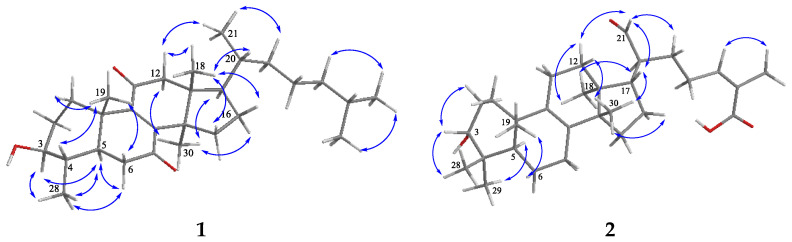
Key NOESY (H↔H) correlations of compounds **1** and **2**.

The relative configuration of **1** was established by a NOESY experiment (Figure 3). NOE correlations between H-3/H-5, H-5/H_3_-28, H_3_-28/H-6*α* and H_3_-30/H-17 and between H_3_-18/H-20, H_3_-18/H-12*β*, H_3_-19/H-4 and H_3_-19/H-6*β* were observed and used to assign their *α* and *β* orientation, respectively. These correlations, as well as the positive optical rotation (${\left[\alpha \right]}_{D}^{20}$ + 68.96°), showed that **1** belongs to the lanostane series [30,31,32].

After detailed analysis of 1D and 2D NMR data (Figure S2) and their comparison with those of 3*β*-hydroxy-4*α*,14*α*-dimethyl-5*α*-ergosta-8,24-diene-7,11-dione [32], compound **1** was determined as 29-norlanost-3*β*-hydroxy-8-ene-7,11-dione, which is a new natural compound.

Compound **2** was isolated as a light yellow solid and identified as 3*α*-hydroxy-tirucall-8,24-dien-21-al-26-oic acid (3-*epi*-isomasticadienolalic acid) by means of 1D and 2D NMR data (Figure S3) and optical rotation. In fact, two identical to **2** structures, differing only in C-20 configuration (C-20 epimers), were previously isolated from *Schinus molle* L. [33,34]. Firstly, in 1978, Pozzo-Balbi et al. [33] characterized the 20S epimer (compound **2**; tirucallane series) and named it 3-*epi*-isomasticadienolalic acid, based on selected ^1^H NMR signals and reactions of reduction and tosylation. Later, Olafsson et al. [34] provided ^13^C NMR data with many interchangeable assignments and claimed that the configuration at C-20 is R (euphol series). In terms of the limited ^1^H NMR data and the fact that euphane and tirucallane triterpenes cannot be distinguished based on ^13^C NMR data, because of very similar carbon resonances [30], we proceeded with detailed ^1^H NMR assignments of **2** (Table 1), and subsequent interpretation of the data of a NOESY experiment. The latter was recognized as an essential approach for discrimination between tirucallanes and euphanes [30,31,35,36,37]. NOE correlations between H-21 and H-16 were reported as characteristic for euphanes and between H-21 and H-12*α* for tirucallanes. For **2**, NOESY correlations between protons of CHO-21/CH_2_-12*α*, CHO-21/CH-17, CH_3_-18/CH-20 and CH_3_-18/CH_2_-12*α* were observed (Figure 2) along with correlations of CH_3_-30/H-17 and CH_3_-30/H12*β* that led to the determination of **2** as a tirucallane. Additionally, compound **2** showed ${\left[\alpha \right]}_{D}^{20}$ − 5.23° (*c* 0.9, CHCl_3_), and negative optical rotation is also associated with tirucallane triterpenes [30]. Similar optical rotation data for a number of tirucallanes, including related 3*α*-tirucallanes, were reported [30,35,37]. In this paper, we report for the first time the ^13^C NMR and detailed ^1^H NMR data of **2** (Table 1).

**Figure 4:**
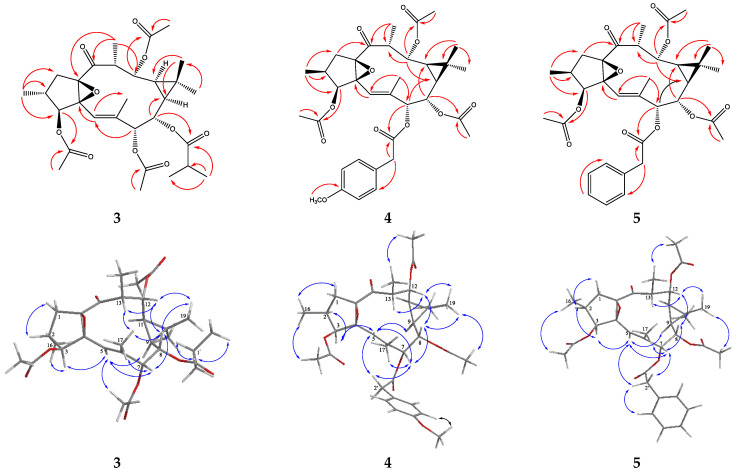
Key HMBC (H→C) and ROESY (H⟷H) correlations of compounds **3**, **4** and **5**.

Compound **3** was isolated as a white amorphous powder in a very low amount. Its HRESIMS showed a sodium adduct ion [M + Na]^+^ at *m*/*z* 585.2667 (Figure S4), corresponding to molecular formula C_30_H_42_O_10_. The ^1^H NMR spectrum, in combination with HSQC data, revealed the presence of five methyl (one vinylic), one methylene and nine methine (one of double bond, and four oxygenated) groups. In addition, a total of four functional groups were distinguished: three acetyl (*δ*_H_ 2.05/*δ*_C_ 20.7, *δ*_H_ 2.10/*δ*_C_ 21.0, and *δ*_H_ 2.15/*δ*_C_ 20.9) and one isobutyryloxy (*δ*_H_ 2.51, sept, *J* = 7.0 Hz, 1H, *δ*_C_ 34.0; *δ*_H_ 1.140, d, *J* = 7.0 Hz, 3H, *δ*_C_ 18.8, and *δ*_H_ 1.138, d, *J* = 7.0 Hz, 3H, *δ*_C_ 18.8), the protons of which showed HMBC correlations with the ester carbonyls at *δ*_C_ 171.0, 170.4, 169.9 and 176.4, respectively (Table 2 and Table 3, Figure 4). These data, along with the chemical shifts and coupling patterns of the CH groups, are in good accordance with those of macrocyclic ditrepenes of ingol type [38,39,40]. Moreover, in the ^1^H NMR spectrum of **3**, the only methylene group CH_2_-1 appeared at *δ*_H_ 2.09 (m, 2H) and *J*_2,3_ is 8.9 Hz (Table 2), which is a characteristic feature for 2-*epi*-ingols [38]. After a detailed comparison of the ^1^H NMR data of **3** with those of 2-*epi*-ingol-3,8,12-triacetate-7-isobutyrate [38], chemical shift differences were detected for the protons of the isopropyl fragment, one of the acetyl group and H-7. For compound **3**, the presence in the ^1^H NMR spectrum of a downshifted acetyl group at *δ*_H_ 2.15, which may be suggested as characteristic of C-7 acetate [39,40,41], together with upshifted signals for the isopropyl protons (~0.1 ppm) was observed; both identical to the position of the isobutyryloxy group at C-8, as it is in 2,3-di*epi*-ingol-7,12-diacetate-8-isobutyrate [38]. It was also confirmed by HMBC correlations of H-3 and H-12 to ester carbonyls at *δ*_C_ 171.0 and 170.4, respectively, as well as by the weak but noticeable correlation of H-8 to C-1′ (*δ*_C_ 176.4).

The relative configuration of **3** was determined by a ROESY experiment. The correlations of H-3 with H_3_-16 and H-5 supported the opposite orientation of H-2 and H-3, while the correlation of H-5 and H-7 was indicative for the *E*-geometry of Δ^5^ [40]. Additionally, the ROESY correlations of H-3/H-5, H-5/H-9 and H-9/H-11 revealed that they are cofacial with assigned *α*-orientation. The correlations of H-7/H-8, H-7/H_3_-17, H-8/H-12, H-8/H-13, H-13/H_3_-17, H_3_-19/H-8 and H_3_-19/H-12 were also observed and assigned to the *β*-orientation of H-7, H-8, H-12, H-13 and CH_3_-19 (Figure 4). All these interactions were consistent with the configuration of ingol diterpenes [40]. Thus, **3** was determined as 2-*epi*-ingol-3,7,12-triacetate-8-isobutyrate (Figures S4 and S5), a new natural compound.

Compound **4** was isolated as a white amorphous powder. Its NMR data were partly similar to those of **3** (Table 2 and Table 3; Figure S6). In the ^1^H NMR spectrum of **4** in CDCl_3_ (Table 2), the signals due to CH_2_-1 appeared at *δ*_H_ 2.78 (1H, dd, *J* = 14.9 and 9.1 Hz) and 1.68 (1H, dd, *J* = 14.9 and 0.9 Hz), which together with *J*_2,3_ = 8.5 Hz was an indication that **4** is a true ingol derivative [38]. The signals for protons of three acetyl groups were also observed, along with signals for a *p*-methoxyphenylacetyl group [*δ*_H_ 7.19 (2H, d, *J* = 8.7 Hz), 6.85 (2H, d, *J* = 8.7 Hz), 3.80 (3H, s) and 3.65 (2H, s)], instead of isobutyryloxy group in **3**. The HMBC correlations of H-8 (*δ*_H_ 4.53, dd, *J* = 10.7 and 1.9 Hz) and H-12 (*δ*_H_ 4.83, dd, *J* = 11.0 and 3.9 Hz) to acetyl carbonyls at *δ*_C_ 170.2 and 170.4, respectively, were used to place two of the acetyl groups at H-8 and H-12. However, due to the small chemical shift differences (0.03 ppm) of H-3 (*δ*_H_ 5.16, d, *J =* 8.5 Hz) and H-7 (*δ*_H_ 5.13, d, *J =* 1.3 Hz), and of the rest two ester carbonyls (0.06 ppm), it was difficult to assign the position of the *p*-methoxyphenylacetyl group and/or of the third acetyl group unambiguously. Moreover, the NMR (^1^H and ^13^C) data of **4** are essentially the same as those of ingol diterpenes with C-3 or C-7 substituted phenylacetyl groups [42,43]. For this reason, the structure of **4** was further elucidated by NMR data recorded in acetone-*d*_6_, where well-shifted signals were observed (Table 2; Figure S7). In acetone-*d*_6_, H-3 and H-7 appeared at *δ*_H_ 5.28 (d, *J* = 8.5 Hz) and *δ*_H_ 5.06 (d, *J* = 1.6 Hz), respectively, and the ^13^C NMR spectrum showed resonances of the four ester carbonyls at *δ*_C_ 170.6, 170.8, 170.9 and 171.1. These data allowed clear assignment of the HMBC correlations, in particular for the position of the *p*-methoxyphenylacetyl group, which was placed at C-7 due to the HMBC cross-peaks of both H-7 and benzylic CH_2_ to the same ester carbonyl at *δ*_C_ 171.1 (C-1′).

The relative configuration of **4** was determined by a ROESY experiment and was identical to that of ingol derivatives [38,40,43]. Cross peaks indicating the opposite orientation of H-2, H-3, H-5, H-9 and H-11 when compared to H-7, H-8, H-12 and H-13 were observed, along with correlation of H-5 and H-7 showing the *E*-geometry of Δ^5^. Additionally, the correlations of H_2_-2′ with both H-5 and H-7, and of H_3_-16/3-acetate and H_3_-20/12-acetate confirmed their position and orientation (Figure 4). Thus, the structure of compound **4** was determined as ingol-7-*p*-methoxyphenylacetyl-3,8,12-triacetate.

Compound **4** was previously isolated from *Euphorbia resinifera* [43,44], but only limited ^1^H NMR data are provided. It was first found and only characterized by Hergenhanh et al. [44] in 1974 on the base of selected ^1^H NMR signals and partial hydrolysis. In this paper, we report for the first time its ^13^C NMR and detailed ^1^H NMR data. 

Compound **5** was isolated as a white amorphous powder. Its 1D and 2D NMR data are highly similar to those of **4** (Table 2 and Table 3; Figure S8). The main difference was the absence of signals for aromatic methoxyl, and thus its 1D NMR spectra showed signals for the monosubstituted aromatic ring at *δ*_H_ 7.26–7.32 and *δ*_C_ 127.8–135.4. Additionally, very small chemical shift differences were observed for H-3 (−0.02 ppm) and H-7 (+0.01 ppm) in CDCl_3_ that resulted in their appearance as overlapping signals at *δ*_H_ 5.14 (d, *J* = 8.5 Hz for H-3 and d, *J* = 1.5 Hz for H-7). In order to follow the HSQC and HMBC correlations of these protons, which also showed the direct H-C correlation to the same carbon resonances (*δ*_C_ 76.9), the NMR spectra of **5** in acetone-*d*_6_ were recorded (Figure S9). In acetone-*d*_6_, the proton and carbon signals for both methine groups were shifted sufficiently and appeared at *δ*_H_ 5.30/*δ*_C_ 77.7 and *δ*_H_ 5.08/*δ*_C_ 77.8, respectively. Further, the HMBC correlations supported the position of two of the acetyl groups at C-8 (acetyl carbonyl at *δ*_C_ 170.8) and C-12 (acetyl carbonyl at *δ*_C_ 170.6). Unfortunately, it was difficult to assign the position of the benzyl and/or the third acetyl group to any of H-3 and H-7 due to extremely close carbon resonances (0.02 ppm) for the rest two ester carbonyls. For this reason, we resorted to the ROESY experiment, where identical to **4** correlations between protons of CH_2_-2′ and both H-5 and H-7, as well as of H_3_-16/3-acetate and H_3_-20/12-acetate allowed assigning of the benzyl group at C-7. All remaining ROESY correlations were also consistent with those of **4**. Thus, compound **5** was determined as ingol-7-phenylacetyl-3,8,12-triacetate.

Similar to **4**, compound **5** was isolated by Hergenhanh et al. [44], and its structure has been elucidated on the base of selected ^1^H NMR signals and partial hydrolysis. In this paper, we report for the first time its ^13^C NMR and detailed ^1^H NMR data.

The remaining isolated compounds were determined as the known 2-*epi*-3,7,12-triacetyl-8-benzoylingol (**6**) [38], lupeol (**7**) [45], 24-methylenecycloartanol (**8**) [46], cycloartanol (**9**) [47], 25-hydroxycycloartanol (**10**) [48], mixture of 29-norcycloartanol (**11**) [49] and obtusifoliol (**12**) (1:0.7) [50], mixture of 6,7-dimethoxy coumarin (scoparon) (**13**) [51] and 6,7,8-trimethoxy coumarin (dimethylfraxetin) (**14**) (1:0.8) [52], and *p*-hydroxybenzoic acid (**15**) [53] based on ^1^H NMR data (Figures S10–S17), and comparison with those in the literature. 

### 2.2. Antimicrobial Activity

The total 70% ethanolic extract and selected isolated compounds were tested for antimicrobial activity against *Staphylococcus aureus* ATCC 29213, *Methicillin-resistant Staphylococcus aureus* (*MRSA*) 1337, *Mycobacterium tuberculosis* ATCC 27294, *Escherichia coli* ATCC 35218 (American Type Cell Culture Collection, Manassas, VA, USA), *Pseudomonas aeruginosa* ATCC 27853, and the fungus *Candida albicans* 562 by broth microdilution method. The results obtained are presented in Table 4.

## 3. Discussion

The detailed chemical analysis of propolis from the semi-arid region of Morocco led to the characterization of 15 compounds, including new lanostane (**1**) and 2-*epi*-ingol (**3**) derivatives. Moreover, seven newly isolated from propolis compounds (**2**, **4**–**6**, and **9**–**11**) were also characterized as the full assignment of one-dimensional (1D) and two-dimensional (2D) nuclear magnetic resonance (NMR) data for three of them (**2**, **4**, and **5**) was reported for the first time. 

Further, suggestions for the botanical origin of the propolis sample analyzed were made based on the comparison between propolis chemistry and the literature data for the plants from which the compounds were previously isolated. The knowledge of the plant sources is, in fact, knowledge of the bees’ choice and preference to certain appropriate resin sources [54], whose availability to the beehives is essential for the wellbeing of the bee colony, as well as for the high quality of propolis [2,55].

All known compounds (except for **2**) were previously reported as constituents of plants in the genus *Euphorbia* [38,44,48,49,56,57,58]. Moreover, the simultaneous presence of triterpenoids, including nortriterpenes (4*α*,14*α*-dimethyl sterols), and macrocyclic diterpenes were detected to a large extent in the areal parts and the latex of *Euphorbia* cactiforms.

The isolated diterpenes are the first macrocyclic diterpenes found in propolis. Until now, ingol diterpenes containing a phenylacetyl group were only shown as constituents of the latex of the endemic to Morocco cactiforms *Euphorbia resinifera* [43,44] and *Euphorbia officinarum* [42]. Among them, diterpenes **4** and **5** were isolated from the latex of *E. resinifera*, while in that of *E. officinarum*, their positional isomers ingol-7,8,12-triacetyl-3-(4-methoxy)phenylacetate and ingol-7,8,12-triacetyl-3-phenylacetate were characterized. *Epi*-ingol derivatives are relatively rare compounds as closely related to **3** structures 2-*epi*-ingol-3,8,12-triacetate-7-isobutyrate and 2,3-di*epi*-ingol-7,12-diacetate-8-isobutyrate are known for *E. portulacoides* [39] and the latex of *E. canariensis* [38], respectively. The latter is also the material from where 2-*epi*-3,7,12-triacetyl-8-benzoylingol (**6**) was previously isolated.

Among the triterpenes, **7**, **8** and **12** are known propolis constituents found in propolis from tropical regions, as the 4*α*,14*α*-dimethyl sterol **12** was only identified in samples from Brazil and Indonesia by gas chromatography–mass spectrometry [59,60,61]. Compounds **2** and **9**–**11** are newly isolated from propolis. Unless **2** was exclusively found in oleoresin of the berries of *Schinus molle* [33], the rest of triterpenoids are common plant metabolites, including for various *Euphorbia* succulents [56,57,62,63]. Obtusifoliol (**12**), together with 31-norlanostenol, for example, are major latex triterpenoids of Moroccan *E. officinarum* L. [64].

Furthermore, looking at the chemistry of studied Moroccan cactiforms, in the latex of the *E. officinarum* L. only triterpenoids and ingol diterpenes were found, until now [42,64,65,66], while that of *E. resinifera* O. Berg is rich in triterpenes, and macrocyclic diterpenes with daphnane, tigliane, ingenane and lathyrane (including ingol derivatives) skeletons [43,56,67]. Based on the chemical comparison between propolis sample analyzed and the literature data of the latex of those species, *E. officinarum* rather than *E. resinifera* could be suggested as a botanical source of the propolis. This is also supported by the fact that *E. resinifera* O. Berg is endemic to the regions of Azilal and Beni Mellal (Middle Atlas) [68], whereas species belonging to the *E. officinarum* group *E. officinarum* L. and *E. officinarum* subsp. *echinus* (Hook.f. and Coss.) Vindt are distributed in the south-western regions of the country from the coast to Anti-Atlas Mountains [69,70]. The propolis sample analyzed was collected from an area of the Sidi-Ifni province (south-western Marocco), where the vegetation is of the infra-Mediterranean type, composed mainly of the succulent *Euphorbia officinarum* subsp. *echinus* (Hook.f. and Coss.) Vind, best known as *Euphorbia echinus* Hook.f. and Coss. [71] (basionym) [63,72,73,74]. This species was also indicated as a source for propolis production by the local beekeepers. Unfortunately, only one article was published on the chemistry of the *E. echinus* latex, focusing on the triterpene composition, and lanostane derivatives were characterized [64].

Although at the current stage of research, we cannot provide unambiguous evidence for a particular cactiform, it is a first scientific insight for the *Euphorbia* spp. as a propolis source. It is also an example of a plant that is a source of materials for both honey [75,76] and propolis production. Only one document where *Euphorbia* spp. is mentioned as a source of propolis was found in the literature. In 2014, Faid [77] revealed the hepatoprotective effect of oil extract of the “Moroccan *Euphorbia resinifera* black propolis” in patients with chronic hepatitis C, but no chemical data or other evidence for the propolis sample tested and/or its botanical source was provided. 

The 70% ethanolic extract and selected isolated compounds were tested in vitro for antimicrobial activity, which is a major driving bioactivity in propolis usage. Moreover, unless the triterpenes are known to possess antimicrobial activity, no data were published for the isolated diterpenes. The results (Table 4) showed that most of the compounds, especially the diterpenes **3**, **4** and **6**, exhibited very good activity against the different strains of bacteria and fungi, compared with the reference antibiotics gentamicin and amphotericin B. Compound **3** is the most active one against all tested bacteria and fungi, with minimum inhibitory concentration (MIC) values in the range of 4–64 µg/mL, and together with cycloartane triterpene **8** is markedly active against *Staphylococcus aureus*, but 2–3-fold less active against *MRSA*. On the whole, most of the compounds exhibited strong antimicrobial activity against *Pseudomonas aeruginosa* and *Candida albicans* as **3**, **8**, **9**, and the mixture of **11** and **12** inhibited *P. aeruginosa* at lower concentrations (MICs 4 μg/mL). All tested compounds (except for **3**; MIC 4 μg/mL) showed moderate activity against the Gram-negative bacteria *Escherichia* *coli* with MICs in the range 16–32 μg/mL and were non-efficient against *Mycobacterium tuberculosis* (MICs ≥ 64 μg/mL). Although the total extract displayed a weaker antibacterial effect against all tested bacteria in comparison to the tested compounds, similar to them appears as more active against the Gram-negative bacteria in comparison to the Gram-positive ones. This is an interesting and promising result considering that propolis from different regions is usually inactive against Gram-negative bacteria [78]. Moreover, taking into consideration that natural products (extracts) usually display MICs in the range 100–1000 μg/mL in the in vitro susceptibility tests, the Moroccan *Euphorbia* propolis can be considered as a promising antimicrobial agent [79] and as a good starting point for further in-depth research of its pharmacokinetics and other relevant properties.

The above-mentioned data are in good accordance with the fact that the latex of *Euphorbia* spp. is known to possess antimicrobial activities, along with other beneficial properties such as antiviral, anti-inflammatory, antiproliferative and cytotoxic activities [56]. This material is widely used in traditional medicine around the world [80,81] as *E. officinarum* and *E. echinus*, for example, are used for the treatment of ophthalmic and various skin diseases [59,63], and the dry latex of *E. resinifera*, called euphorbium, which is also commercially available in many countries, is used for the treatment of neurological problems, chronic pain, tuberculosis, etc. [82]. On the other hand, however, it should be mentioned that along with the beneficial properties, the *Euphorbia* latex is well known for its toxic and irritant effect on the skin and mucous membranes, and the requirements for usage of low quantities were underlined [83]. The adverse effects have been attributed to macrocyclic diterpenes in a great majority of tigliane (phorbol esters), daphnane and ingenane types [44,83,84,85]. For the ingol diterpenes ingol-7-*p*-methoxyphenylacetyl-3,8,12-triacetate (**4**) and ingol-7-*p*-methoxyphenylacetyl-3,8,12-triacetate (**5**), in particular, Hergenhahn et al. [44,84] reported that they could be considered practically inactive as irritants, a conclusion based on studies on a mouse ear.

## 4. Materials and Methods

### 4.1. General Experimental Procedures

Optical rotations were measured with a Jasco P-2000 polarimeter. Nuclear magnetic resonance (NMR) spectra were recorded on a Bruker AVANCEII + 600 NMR spectrometer operating at 600 MHz (150 MHz for ^13^C). High-resolution electrospray ionization mass spectra (HRESIMS) were obtained on Thermo Scientific Q Extractive Plus Mass spectrometer with Orbitrap Analyser. Vacuum liquid chromatography (VLC) was performed on Silica gel 60H (15 μm, Merck, Darmstadt, Germany). Low-pressure liquid chromatography (LPLC) was carried out on LiChroprep Si 60 Merck column (40–63 μm). Column chromatography (CC) was performed on Silica gel 60 (63–200 μm, Merck), silver nitrate-impregnated silica gel (~10 wt.% loading, 230 mesh, Sigma-Aldrich, St. Louis, MO, USA) and Sephadex LH-20 (25–100 μm, Pharmacia Fine Chemicals, Uppsala, Sweden). Preparative thin-layer chromatography (PTLC) was performed on silica gel 60F_254_ glass plates (20 × 20 cm, 0.25 mm, Merck). Detection of the spots was achieved under UV light at 254 and 366 nm, and subsequently spraying with vanillin in sulfuric acid and heating at 100 °C. All solvents used were of analytical grade.

### 4.2. Propolis Sample

The propolis sample was collected by scraping from *Apis mellifera* bees’ hives in Sidi-Ifni province (Guelmin-OuedNoun region) in Morocco in August 2018.

### 4.3. Extraction and Isolation

The raw propolis (40.0 g) was extracted with 70% ethanol (1:10, *w*/*v*) at room temperature (2 × 24 h). The extracts obtained were filtrated, combined and concentrated on a rotary evaporator. The total extract was suspended in water and subjected to liquid–liquid extraction successively with petroleum ether (PE, 3 times) and chloroform (CHCl_3_, 3 times) to provide 4.5 g and 3.6 g dry residue, respectively.

The PE extract (4.3 g) was then subjected to silica gel VLC, eluted with a gradient system of PE:EtOAc (1:0 to 0:1), to obtain twelve fractions A–L.

Fraction E (424.7 mg) was separated by LPLC using a gradient system of hexane:EtOAc (95:5 to 0:1) to provide thirty two fractions (E1–E32). Fractions E10 (66.6 mg) and E11 (75.8 mg) were subjected individually on silver nitrate-impregnated silica gel CC with hexane:CH_2_Cl_2_ (98:2 to 0:1) as a mobile phase, and fourteen (E10.1–E10.14) and seventeen (E11.1–E11.17) subfractions were obtained, respectively. Subfraction E10.2 yielded cycloartanol (**9**) (3.4 mg). Subfractions E11.12 and E11.15 yielded lupeol (**7**) (13.1 mg) and 24-methylenecycloartanol (**8**) (5.0 mg), respectively. Fraction E15 (16.0 mg) after purification by PTLC with PE:Et_2_O (6:4) as a mobile phase afforded an inseparable mixture of 29-norcycloartanol (**11**) and obtusifoliol (**12**) (5.5 mg). Fraction G (609.8 mg) was subjected to Sephadex LH-20 CC, eluted with CHCl_3_:CH_3_OH (1.5:1), and nine combined fractions (G1–G9) were obtained. Fraction G2 (123.9 mg), after additional separation by silica gel CC, eluted with hexane:Et_2_O (1:0 to 0:1), afforded nine subfractions (G2.1–G2.9). Subfractions G2.2 and G2.3 were combined (9.4 mg) and purified by PTLC with PE:Et_2_O (6:4) to yield 2-*epi*-ingol-3,7,12-triacetate-8-isobutyrate (**3**) (0.8 mg) and 2-*epi*-3,7,12-triacetyl-8-benzoylingol (**6**) (2.8 mg). After purification by PTLC with PE:Et_2_O (6:4), subfraction G2.6 (12.3 mg) yielded ingol-7-phenylacetyl-3,8,12-triacetate (**5**) (7.7 mg). Subfraction G2.8 yielded ingol-7-*p*-methoxyphenylacetyl-3,8,12-triacetate (**4**) (34.8 mg). Fraction H (678.3 mg) was subjected to LPLC using CHCl_3_:EtOAc (1:0 to 0:1) as a mobile phase and twenty fractions (H1–H20) were obtained. After additional purification by PTLC, eluted with CHCl_3_:MeOH (20:1), fraction H15 (30.4 mg) afforded 29-norlanost-3*β*-hydroxy-8-ene-7,11-dione (**1**) (2.4 mg) and 25-hydroxycycloartanol (**10**) (4.6 mg).

The CHCl_3_ extract (3.0 g) was subjected to silica gel VLC eluted with a gradient system of CH_2_Cl_2_:EtOAc (1:0 to 0:1). Seven fractions were obtained A′-G′. Fraction B′ (165.0 mg) was separated by LPLC with CHCl_3_:EtOAc (97:3 to 0:1) as a mobile phase, and eight fractions were obtained (B′1-B′8). Fraction B′2 (41.0 mg) was purified by PTLC with PE:EtOAc (7:3) to yield a mixture of 6,7-dimethoxy coumarin (scoparon) (**13**) and 6,7,8-trimethoxy coumarin (dimethylfraxetin) (**14**) (1.4 mg). Fraction D′ (615.2 mg) was subjected to Sephadex LH-20 CC, eluted with CH_3_OH, and five fractions were obtained D′1-D′5. Fraction D′5 yielded *p*-hydroxybenzoic acid (**15**) (20.4 mg). Fraction E′ (381.5 mg) was also subjected to Sephadex LH-20 CC, eluted with CH_3_OH, and five fractions were obtained E′1–E′5. Fractions D′2 (193.4 mg), D′3 (244.3 mg), E′3 (185.0 mg) and E′4 (23.1 mg) were combined and after silica gel CC, eluted with CHCl_3_:Acetone (98:2 to 0:1), and further rechromatography with LPLC using Et_2_O:CHCl_3_ (7:3) as a mobile phase 3*α*-hydroxy-tirucall-8,24-dien-21-al-26-oic acid (**2**) (8.3 mg) was yielded.

#### 4.3.1. 29-Norlanost-3*β*-hydroxy-8-ene-7,11-dione (**1**)

White amorphous powder; ${\left[\alpha \right]}_{D}^{20}$ + 68.96° (*c* 0.16, CHCl_3_); ^1^H and ^13^C NMR data, see Table 1; HRESIMS *m*/*z* 443.3527 [M + H]^+^ (calcd for C_29_H_47_O_3_, 443.3525).

#### 4.3.2. 2-*Epi*-ingol-3,7,12-triacetate-8-isobutyrate (**3**)

White amorphous powder; ^1^H and ^13^C NMR data, see Table 2 and Table 3; HRESIMS *m*/*z* 585.2667 [M + Na]^+^ (calcd for C_30_H_42_O_10_Na, 585.2676).

### 4.4. Antimicrobial Activity

#### 4.4.1. Test Microorganisms

For antimicrobial activity, the following test-microorganisms were used: *Escherichia coli* ATCC 35218 (American Type Cell Culture Collection, Manassas, VA, USA), *Staphylococcus aureus* ATCC 29213, *Methicillin-resistant Staphylococcus aureus* (*MRSA*) 1337, *Pseudomonas aeruginosa* ATCC 27853, *Mycobacterium tuberculosis* ATCC 27294, and the fungus *Candida albicans* 562 from the SAIM-BAS collection.

#### 4.4.2. Culture Medium and Growth Conditions

The cultivation of *P. aeruginosa* and *C. albicans* was performed on Brain Heart Infusion Broth and Agar (BHIB, GM210, resp. BHIA, M1611, HiMedia Laboratories, GmbH, Einhausen, Germany), *S. aureus* and *E. coli* on Mueller Hinton Agar and Broth (MHA, M173, resp. MHB, M391, HiMedia Laboratories, Germany) at 37 °C for 18 h. *M. tuberculosis* were cultured in Lowestein–Jensenn and Middlebrook 7H9 medium, HiMedia Laboratories, Germany, at 37 °C until log phase growth.

#### 4.4.3. Minimal Inhibitory Concentration (MIC)

The in vitro antimicrobial activity of the total extract and selected isolated compounds was determined by the broth microdilution method according to ISO 20776-1:2006 [86]. Briefly, the bacterial inoculums with concentration 10^5^ CFU/mL were added to 96-well plates containing MHB or BHIB loaded with two-fold serial dilutions of the tested samples. Plates were incubated at 37 °C for 18 h. According to EUCAST requirements, gentamicin for the test bacteria and amphotericin B for *C. albicans* were used. Experiments were performed in triplicate. In vitro antimycobacterial activity was assessed according to the EUCAST broth microdilution reference method for MIC determination [87]. Briefly, bacterial suspension was prepared at a concentration of about 2 × 10^6^ cells/mL and further diluted 1:20 in Middlebrook 7H9 medium with 10% OADC (oleic acid–albumin–dextrose–catalase) (Becton Dickinson and Co., Sparks, MD, USA). Ninety-six-well microplates were used in which Middlebrook 7H9 medium was added dropwise with the appropriate concentration of test compounds range 0.125 µg/mL to 512 µg/mL and *M. tuberculosis* suspension. Ethambutol and isoniazid were used as controls. Reading was performed after 7, 14, and 21 days incubation at 37 °C using an inverted mirror. The MIC was the lowest concentration without visual growth and was expressed as µg/mL.

## 5. Conclusions

The detailed chemical analysis and antimicrobial evaluation of propolis from a semi-arid region of Morocco were performed for the first time. The results revealed that it possesses specific chemical composition with triterpenoids and ingol diterpenes as characteristic and antimicrobial compounds. *Euphorbia* spp., most probably *Euphorbia officinarum* subsp. *echinus*, could be suggested as a plant source of the propolis. Further studies are needed in order to prove the particular botanical source, as well as to reveal the area of distribution of this specific propolis type. Special attention should be paid in respect to the possibilities for its application and safety use.

## Figures and Tables

**Table 1:** ^1^H and ^13^C NMR data for **1** and **2** in CDCl_3_ (^1^H at 600 MHz, ^13^C at 150 MHz, *δ* in ppm, *J* in Hz).

	1		2	
	^1^H	^13^C	^1^H	^13^C ^b^
1	2.84 dt (13.5, 3.6) 1.15 dd (13.5, 3.8)	33.3	1.51 m 1.25 m	29.7
2	1.89 m 1.62 m	30.9	1.93 m 1.61 m	25.7
3	3.13 ddd (11.2, 9.7, 5.1)	75.4	3.43 brs	75.9
4	1.53 m	38.1	-	37.6
5	1.45 m	47.1	1.58 m	44.7
6	2.51 dd (15.4, 2.7) 2.29 t (15.1)	38.2	1.58 m 1.40 m	18.7
7	-	201.6	2.06 m 1.94 m	27.1
8	-	151.3	-	132.7
9	-	151.5		134.6
10	-	38.6	-	37.2
11	-	202.8	2.03 m 1.94 m	21.2
12	2.76 dd (16.0, 0.9) 2.64 brd (16.0)	51.5	1.58 m 1.28 m	29.5
13	-	47.5	-	43.9
14	-	49.0	-	49.6
15	2.13 ddd (13.9, 11.8, 2.8) 1.72 dt (11.8, 7.2)	32.1	1.58 m 1.47 m	29.5
16	1.98 m 1.34 m	27.3	1.93 m 1.40 m	26.6
17	1.66 m	49.1	2.06 m	45.2
18	0.81 s	16.8	0.77 s	16.7
19	1.30 s	16.4	0.94 s	19.9
20	1.43 m	36.2 ^a^	2.28 m	55.3
21	0.88 d (6.5)	18.6	9.51 d (5.5)	206.1
22	1.00 m	36.2 ^a^	1.64 m	28.6
23	1.36 m 1.14 m	24.0	2.42 m	27.5
24	1.13 m	39.4	6.04 td (7.7, 1.4)	144.6
25	1.51 m	28.0	-	127.1
26	0.86 d (6.6)	22.5	-	172.3
27	0.87 d (6.6)	22.8	1.91 s	20.5
28	1.01 d (6.3)	14.8	0.96 s	28.1
29	-	-	0.85 s	22.2
30	1.18 s	26.0	0.88 s	24.3

^a^ 36.16 for C-20; 36.19 for C-22; ^b^ The assignments were based on HSQC and HMBC data.

**Table 2:** ^1^H NMR data of compounds **3**–**5** at 600 MHz (*δ* in ppm, *J* in Hz).

	3	4		5	
	CDCl_3_	CDCl_3_	Acetone-*d*_6_	CDCl_3_	Acetone-*d*_6_
1*α* 1*β*	2.09 m	2.78 dd (14.9, 9.1) 1.68 dd (14.9, 0.9)	2.76 dd (14.8, 9.0) 1.66 dd (14.8, 0.7)	2.77 dd (14.9, 9.0) 1.68 dd (14.9, 0.9)	2.78 dd (14.8, 9.0) 1.68 dd (14.8, 0.9)
2	1.89 m	2.50 m	2.49 m	2.50 m	2.49 m
3	5.04 d (8.9)	5.16 d (8.5)	5.28 d (8.5)	5.14 d (8.5)	5.30 d (8.5)
4	-	-	-	-	-
5	5.62 brs	5.40 br s	5.65 br s	5.39 br s	5.69 br s
6	-	-	-	-	-
7	4.98 d (2.1)	5.13 d (1.3)	5.06 d (1.6)	5.14 d (1.5)	5.08 d (1.5)
8	4.58 dd (11.0, 2.1)	4.53 dd (10.7, 1.9)	4.64 dd (10.8, 1.9)	4.53 dd (10.7, 1.9)	4.66 dd (10.8, 1.9)
9	1.32 dd (10.7, 9.0)	1.12 dd (10.6, 9.2)	1.28 dd (10.8, 9.1)	1.10 dd (10.4, 9.1)	1.29 dd (10.8, 9.1)
10	-	-	-	-	-
11	1.00 m	1.05 overlapping	1.04 dd (9.1, 2.0)	1.03 m	1.05 dd (9.3, 1.9)
12	4.86 dd (11.0, 3.8)	4.83 dd (11.0, 3.9)	4.90 dd (11.1, 4.0)	4.83 dd (11.0, 3.8)	4.91 dd (11.1, 4.0)
13	2.93 m	2.88 dq (7.3, 4.0)	3.00 dq (7.2, 4.0)	2.88 dq (7.2, 4.4)	3.01 dq (7.2, 4.0)
14	-	-	-	-	-
15	-	-	-	-	-
16	1.04 ^a^ d (6.8)	0.92 d (7.5)	0.91 d (7.5)	0.92 d (7.5)	0.92 d (7.5)
17	2.07 d (0.9)	2.06 d (1.2)	2.08 d (1.3)	2.07 d (1.4)	2.09 d (1.3)
18	1.11 s	1.05 ^c^ s	1.02 s	1.05 s	1.03 s
19	0.84 s	0.82 s	0.82 s	0.82 s	0.83 s
20	1.04 ^a^ d (7.3)	1.05 ^c^ d (7.3)	0.99 d (7.3)	1.05 d (7.2)	1.00 d (7.3)
1′	-	-	-	-	-
2′	2.51 sept (7.0)	3.65 s	3.62, 3.66 AB q (15.3)	3.72 s	3.71, 3.75, AB q (15.3)
3′	1.14 ^b^ d (7.0)	-	-	-	-
4′	1.14 ^b^ d (7.0)	7.19 d (8.7)	7.22 d (8.7)	7.28 m	7.32 m
5′, 7′	-	6.85 d (8.7)	6.86 d (8.7)	7.31 m	7.32 m
6′	-	-	-	7.26 m	7.26 m
8′	-	7.19 d (8.7)	7.22 d (8.7)	7.28 m	7.32 m
OCH_3_	-	3.80 s	3.77 s	-	-
3-OAc	2.05 s	2.06 s	2.00 s	2.06 s	2.01 s
7-OAc	2.15 s	-	-	-	
8-OAc	-	1.97 s	1.94 s	1.97 s	1.94 s
12-OAc	2.10 s	2.09 s	2.01 s	2.09 s	2.02 s

^a^ 1.035 for CH_3_-16 and 1.040 for CH_3_-20; ^b^ 1.140 for CH_3_-3′ and 1.138 CH_3_-4′; ^c^ 1.054 for CH_3_-18 and 1.046 for CH_3_-20.

**Table 3:** ^13^C NMR data of compounds **3**–**5** at 150 MHz (*δ* in ppm).

	3	4		5	
	CDCl_3_	CDCl_3_	Acetone-*d*_6_	CDCl_3_	Acetone-*d*_6_
1	31.0	31.4	32.1	31.4	32.1
2	31.1	29.5	30.4	29.4	30.3
3	80.4	76.7	77.7	76.9	77.7
4	71.1	73.3	74.3	73.3	74.3
5	117.1	117.0	118.7	117.1	118.8
6	139.3	139.3	139.8	139.2	139.7
7	76.8	76.8	77.8	76.9	77.8
8	70.8	71.4	72.1	71.4	72.1
9	24.6	24.6	25.8	24.6	25.8
10	19.1	19.2	19.8	19.2	19.8
11	30.6	30.6	31.7	30.6	31.7
12	70.9	70.6	71.3	70.6	71.3
13	42.9	43.0	43.7	43.0	43.7
14	207.3	207.6	207.3	207.6	207.3
15	71.1	71.1	71.9	71.0	72.0
16	16.1	16.9	17.3	16.9	17.3
17	17.3	17.4	17.7	17.4	17.7
18	29.1	29.0	29.4	29.0	29.4
19	16.3	16.0	16.6	16.0	16.6
20	13.4	13.4	13.7	13.4	13.7
1′	176.4	170.6 ^a,b^	171.1	170.6 ^a,c^	170.9 ^a,d^
2′	34.0	40.5	40.9	41.5	41.8
3′	18.8	125.8	127.3	133.8	135.4
4′	18.8	130.3	131.3	129.3	130.4
5′, 7′	-	114.0	114.7	128.6	129.3
6′	-	158.7	159.8	127.2	127.8
8′	-	130.3	131.3	129.3	130.4
OCH_3_	-	55.2	55.5	-	-
3-OAc	20.7 171.0	20.5 170.6 ^a,b^	20.5 170.9	20.6 170.3 ^a,c^	20.5 170.8 ^a,d^
7-OAc	20.9 169.9	-	-	-	-
8-OAc	-	20.9 170.2	20.9 170.8	20.9 170.3	21.0 170.8 ^e^
12-OAc	21.0 170.4	21.0 170.4	21.0 170.6	21.00 170.4	21.0 170.6

^a^ Interchangable signals within the column; ^b^ 170.64/170.58; ^c^ 170.63/170.29; ^d^ 170.86/170.84; ^e^ 170.79.

**Table 4:** Antibacterial and antifungal activities of the total 70% ethanolic extract and isolated compounds.

Samples	Minimum Inhibitory Concentration (MIC) (µg/mL)					
	Gram-Positive Bacteria			Gram-Negative Bacteria		Fungi
	*S. aureus* 29213	MRSA 1337	*M. tuberculosis* H37Rv ATCC 27294	*E. coli* 35218	*P. aeruginosa* ATCC 27853	*C. albicans* 562
70% ethanolic extract	128	128	>512	64	64	32
**1**	64	64	512	32	16	4
**2**	64	64	128	32	32	32
**3**	4	16	64	4	4	4
**4**	32	32	64	16	16	8
**5**	32	64	128	32	32	8
**6**	32	32	64	16	8	8
**7**	32	64	128	16	16	4
**8**	16	64	64	16	4	4
**9**	64	64	128	16	4	4
**11** and **12** (1:0.7)	32	64	128	16	4	4
Gentamicin ^a^	0.5	0.25	NT	0.5	2	NT
Amphotericin B ^a^	NT	NT	NT	NT	NT	0.125
Isoniazid ^a^	NT	NT	0.25	NT	NT	NT
Ethambutol ^a^	NT	NT	8	NT	NT	NT

NT—Not tested; ^a^ Positive control.

## Data Availability

The data presented in this study are available upon request from the corresponding author.

## Supplementary Materials

The following materials are available online at https://www.mdpi.com/article/10.3390/molecules27072206/s1, Figure S1. HRESIMS spectrum of **1**; Figure S2. ^1^H NMR, ^13^C NMR, ^1^H-^1^H COSY, HSQC, HMBC and NOESY spectra of **1** in CDCl_3_; Figure S3. ^1^H NMR, ^13^C NMR, ^1^H-^1^H COSY, HSQC, HMBC and NOESY spectra of **2** in CDCl_3_; Figure S4. HRESIMS spectrum of **3**; Figure S5. ^1^H NMR, ^1^H-^1^H COSY, HSQC, HMBC and ROESY spectra of **3** in CDCl_3_; Figure S6. ^1^H NMR, ^13^C NMR, DEPT, ^1^H-^1^H COSY, HSQC, HMBC and ROESY spectra of **4** in CDCl_3_; Figure S7. ^1^H NMR, ^13^C NMR, HSQC and HMBC spectra of **4** in acetone-*d*_6_; Figure S8. ^1^H NMR, ^13^C NMR, HSQC, HMBC and ROESY spectra of **5** in CDCl_3_; Figure S9. ^1^H NMR, ^13^C NMR, HSQC, HMBC and ROESY spectra of **5** in acetone-*d*_6_; Figure S10. ^1^H NMR spectrum of **6** in CDCl_3_; Figure S11. ^1^H NMR spectrum of **7** in CDCl_3_; Figure S12. ^1^H NMR spectrum of **8** in CDCl_3_; Figure S13. ^1^H NMR spectrum of **9** in CDCl_3_; Figure S14. ^1^H NMR spectrum of **10** in CDCl_3_; Figure S15. ^1^H NMR spectrum of a mixture of **11** and **12** (1:0.7) in CDCl_3_; Figure S16. ^1^H NMR spectrum of a mixture of **13** and **14** (1:0.8) in CDCl_3_; Figure S17. ^1^H NMR spectrum of **15** in CDCl_3_:CD_3_OD (1:1).

## Sample Availability

Samples are available upon reasonable request from the corresponding author.
