# Supplementary material for: Antimicrobial Triterpenoids and Ingol Diterpenes from Propolis of Semi-Arid Region of Morocco
Source: Molecules. 2022 Mar 28;27(7):2206. doi: 10.3390/molecules27072206 (PMC9000693; doi:10.3390/molecules27072206)

Supplementary material

Antimicrobial triterpenoids and ingol diterpenes from propolis of semi-arid region of Morocco

Ralitsa Chimshirova[1], Milena Popova[1*], Amina Chakir[2], Violeta Valcheva[3], Simeon Dimitrov[3], Boryana Trusheva[1], Abderrahmane Romane[2], Vassya Bankova[1]

[1]Institute of Organic Chemistry with Centre of Phytochemistry, Bulgarian Academy of Sciences, Acad. G. Bonchev str., bl. 9, 1113, Sofia, Bulgaria.
[2]Laboratory of Applied Chemistry, Faculty of Sciences Semlalia, Cadi Ayyad University, Boulevard Prince My Abdellah B.P. 2390, 40000, Marrakech, Morocco.
[3]The Stephan Angeloff Institute of Microbiology, Department of Infectious microbiology, Bulgarian Academy of Sciences, Acad. G. Bonchev st., bl. 26, Sofia, Bulgaria.

[*]Correspondence: Milena.Popova@orgchm.bas.bg

Content

Figure S1. HRESIMS spectrum of **1**……………………………………………………..3
Figure S2. ^{1}H NMR, ^{13}C NMR, ^{1}H-^{1}H COSY, HSQC, HMBC and NOESY spectra of **1** in $CDCl_3$……………………………………………………………………….4
Figure S3. ^{1}H NMR, ^{13}C NMR, ^{1}H-^{1}H COSY, HSQC, HMBC and NOESY spectra of **2** in $CDCl_3$……………………………………………………………………….7
Figure S4. HRESIMS spectrum of **3**……………………………………………………..10
Figure S5. ^{1}H NMR, ^{1}H-^{1}H COSY, HSQC, HMBC and ROESY spectra of **3** in $CDCl_3$...11
Figure S6. ^{1}H NMR, ^{13}C NMR, DEPT, ^{1}H-^{1}H COSY, HSQC, HMBC and ROESY spectra of **4** in $CDCl_3$……………………………………………………………………….14
Figure S7. ^{1}H NMR, ^{13}C NMR, HSQC and HMBC spectra of **4** in acetone-d_6…………18
Figure S8. ^{1}H NMR, ^{13}C NMR, HSQC, HMBC and ROESY spectra of **5** in $CDCl_3$……20
Figure S9. ^{1}H NMR, ^{13}C NMR, HSQC, HMBC and ROESY spectra of **5** in acetone-d_6…………………………………..……………………………………………23
Figure S10. ^{1}H NMR spectrum of **6** in $CDCl_3$………………………………………………26
Figure S11. ^{1}H NMR spectrum of **7** in $CDCl_3$………………………………………………26
Figure S12. ^{1}H NMR spectrum of **8** in $CDCl_3$………………………………………………27
Figure S13. ^{1}H NMR spectrum of **9** in $CDCl_3$………………………………………………27
Figure S14. ^{1}H NMR spectrum of **10** in $CDCl_3$……………………………………………..28
Figure S15. ^{1}H NMR spectrum of a mixture of **11** and **12** (1:0.7) in $CDCl_3$……………..28
Figure S16. ^{1}H NMR spectrum of a mixture of **13** and **14** (1:0.8) in $CDCl_3$……………..29
Figure S17. ^{1}H NMR spectrum of **15** in $CDCl_3$:CD_3OD (1:1)……………………………..29

Figure S1. HRESIMS spectrum of **1**.

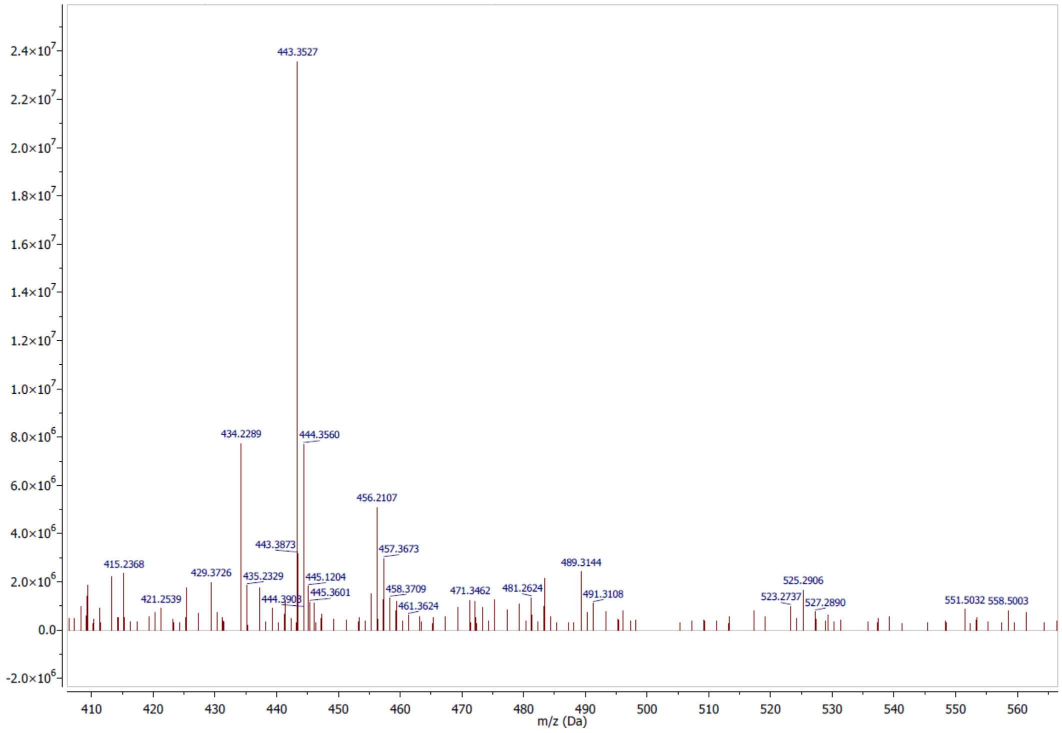

Figure S2. ^{1}H NMR, ^{13}C NMR, ^{1}H - ^{1}H COSY, HSQC, HMBC and NOESY spectra of **1** in $CDCl_3$.

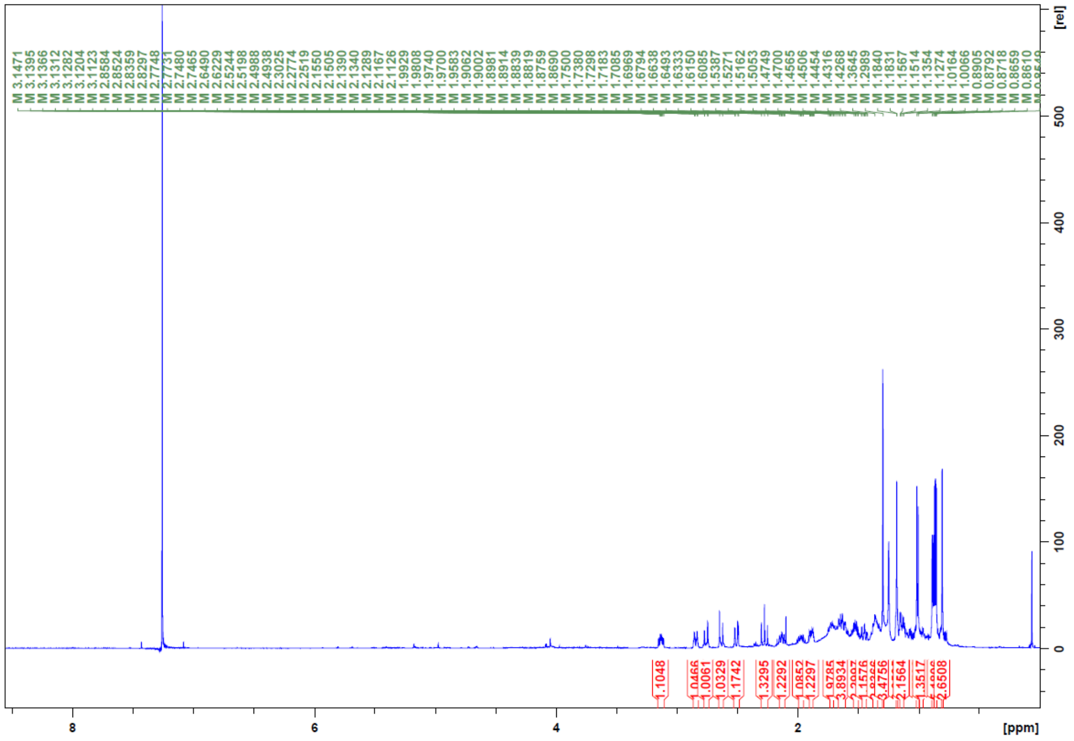

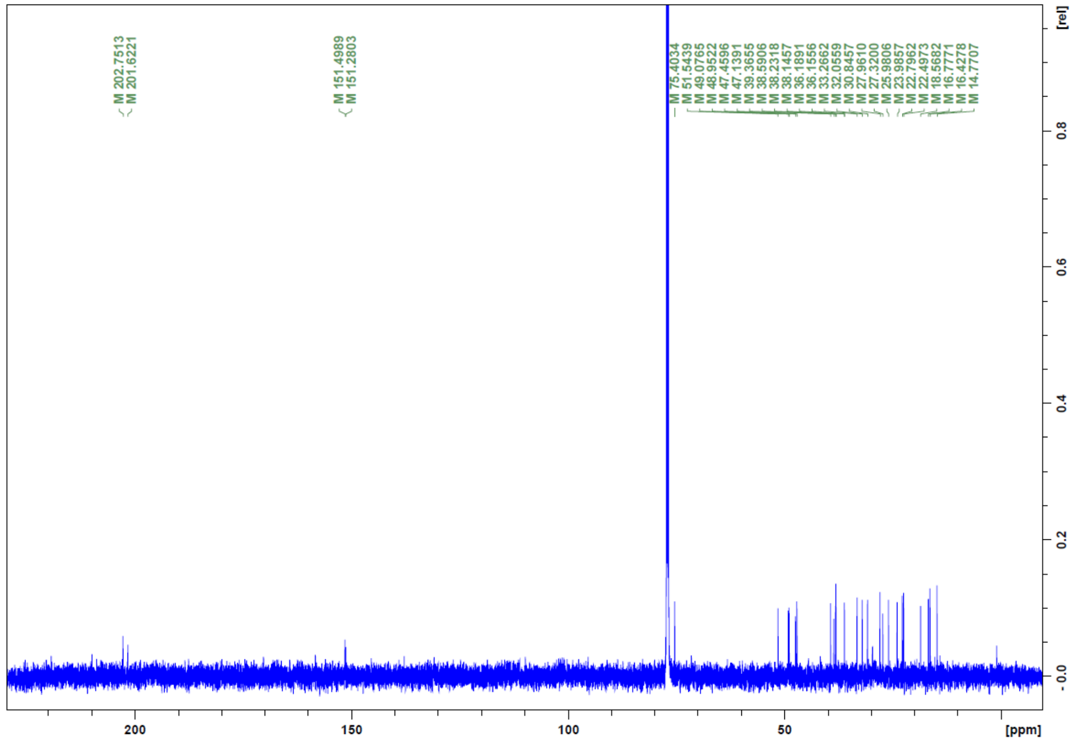

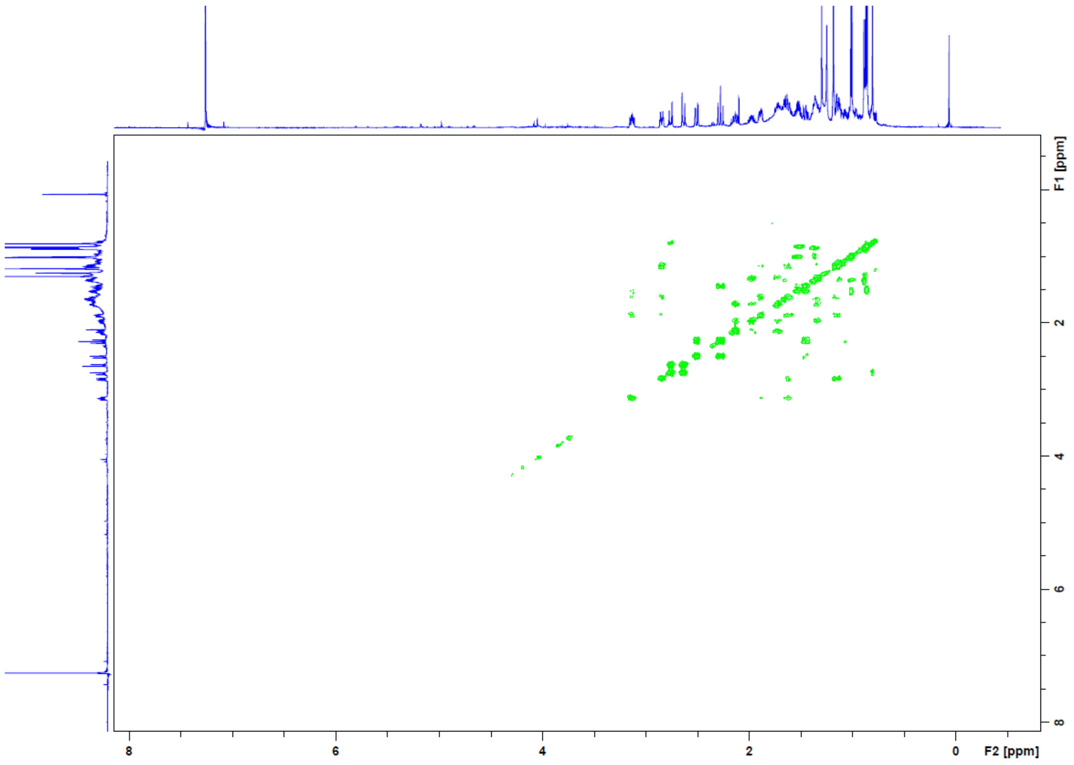
F1 [ppm]
2
4
6
8
8
6
4
2
0
F2 [ppm]

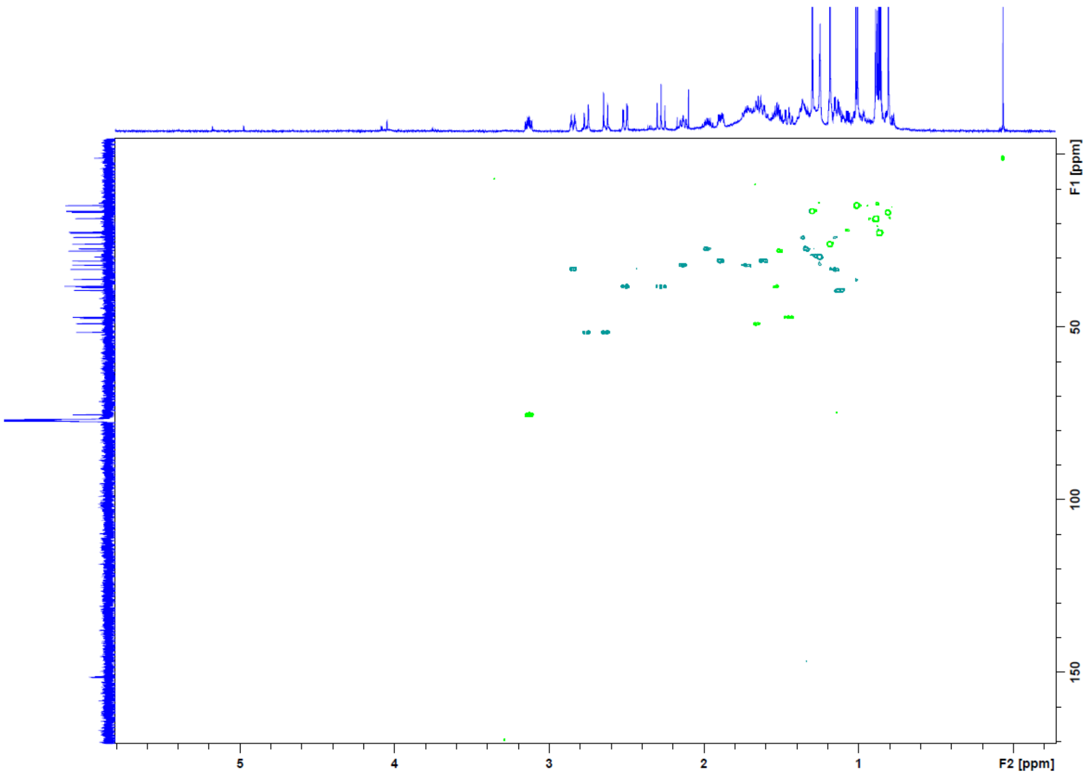
F1 [ppm]
50
100
150
5
4
3
2
1
F2 [ppm]

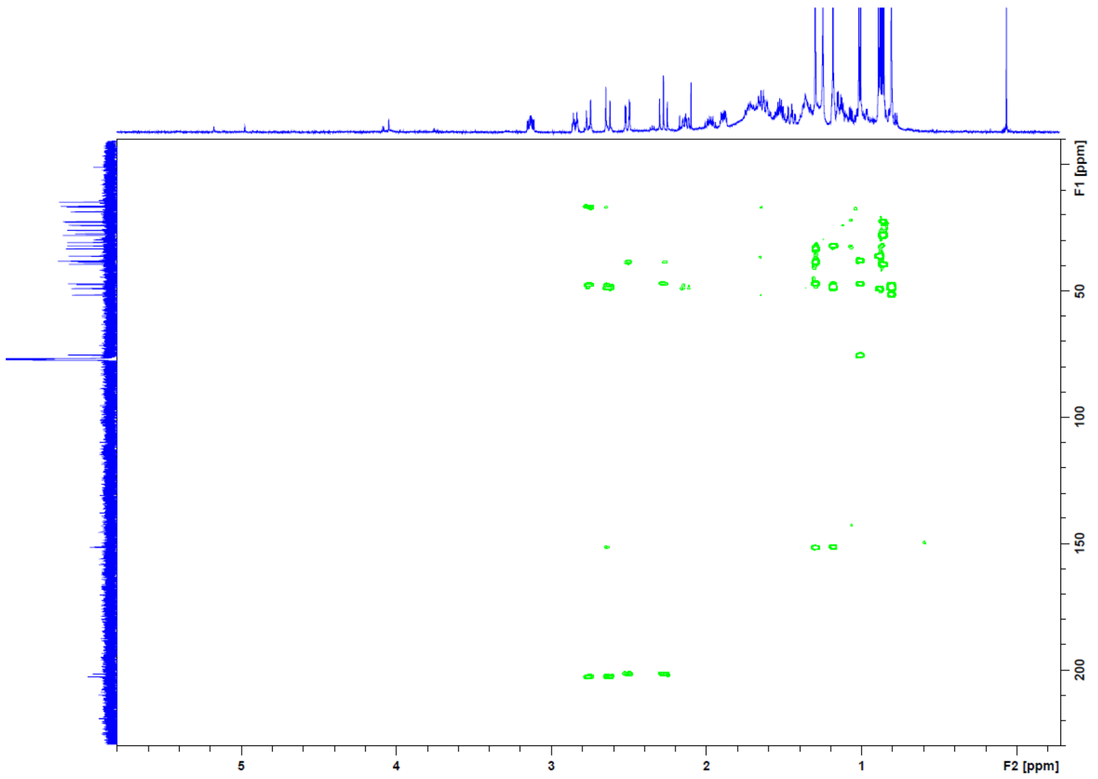
F1 [ppm]
50
100
150
200
5
4
3
2
1
F2 [ppm]

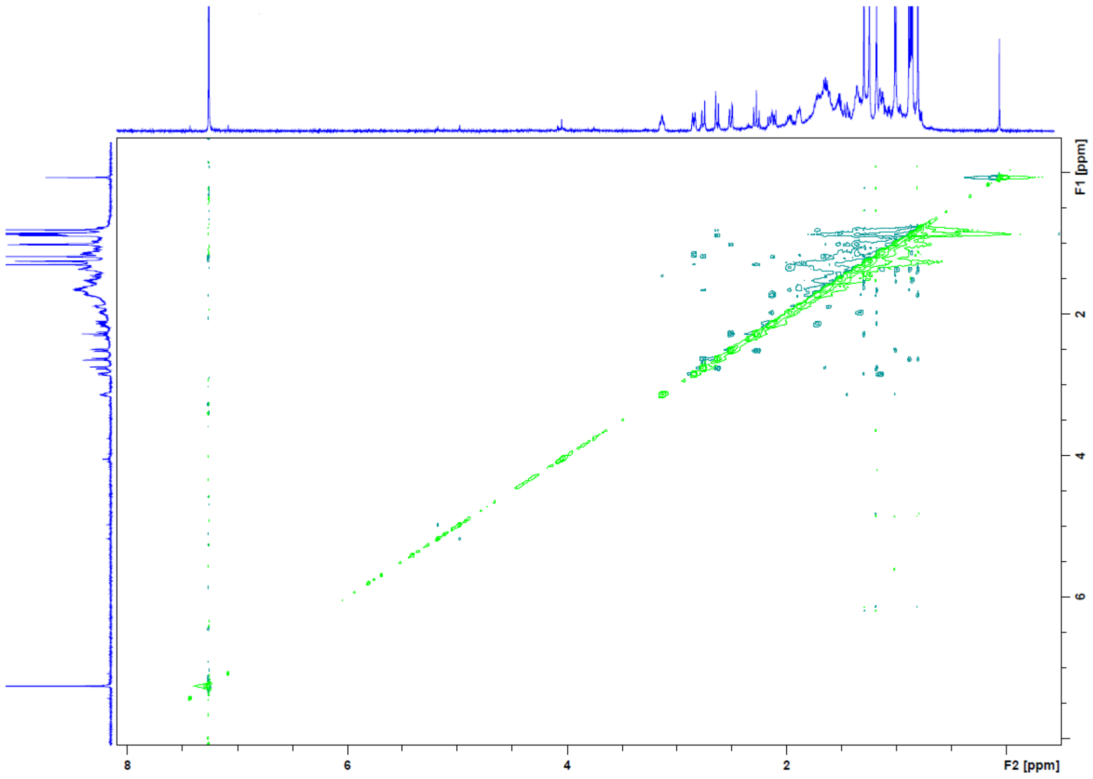
F1 [ppm]
2
4
6
8
6
4
2
F2 [ppm]

Figure S3. ^{1}H NMR, ^{13}C NMR, ^{1}H - ^{1}H COSY, HSQC, HMBC and NOESY spectra of **2** in $CDCl_3$.

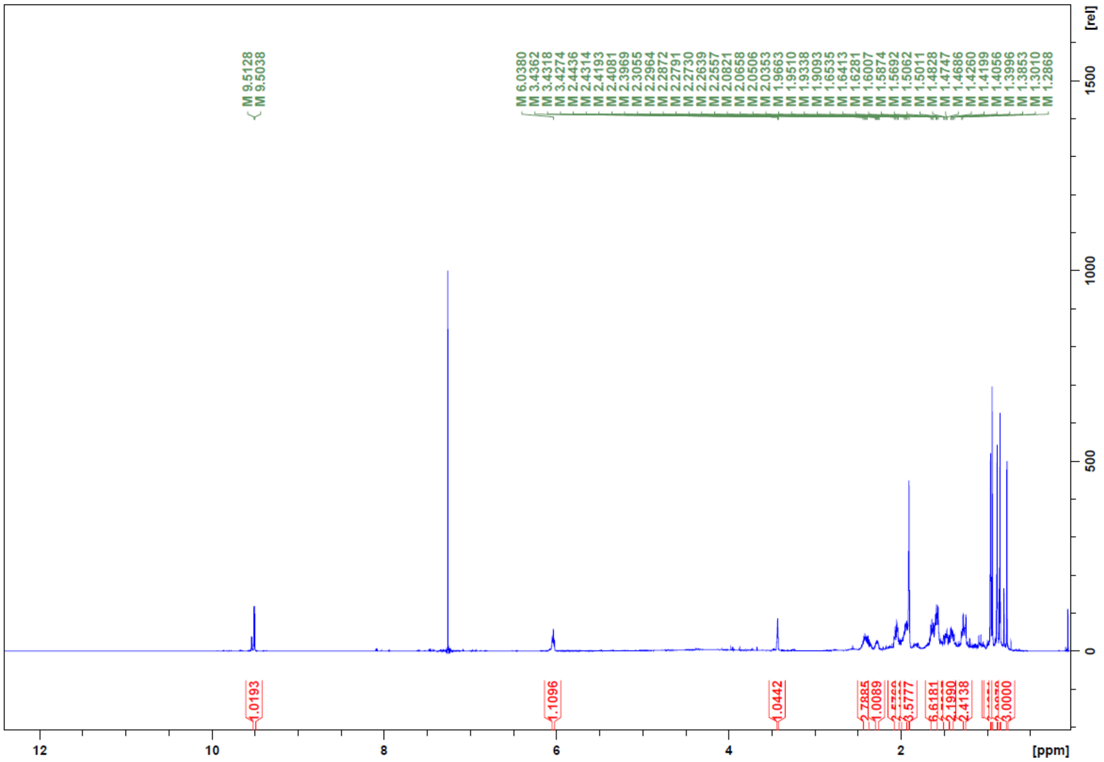

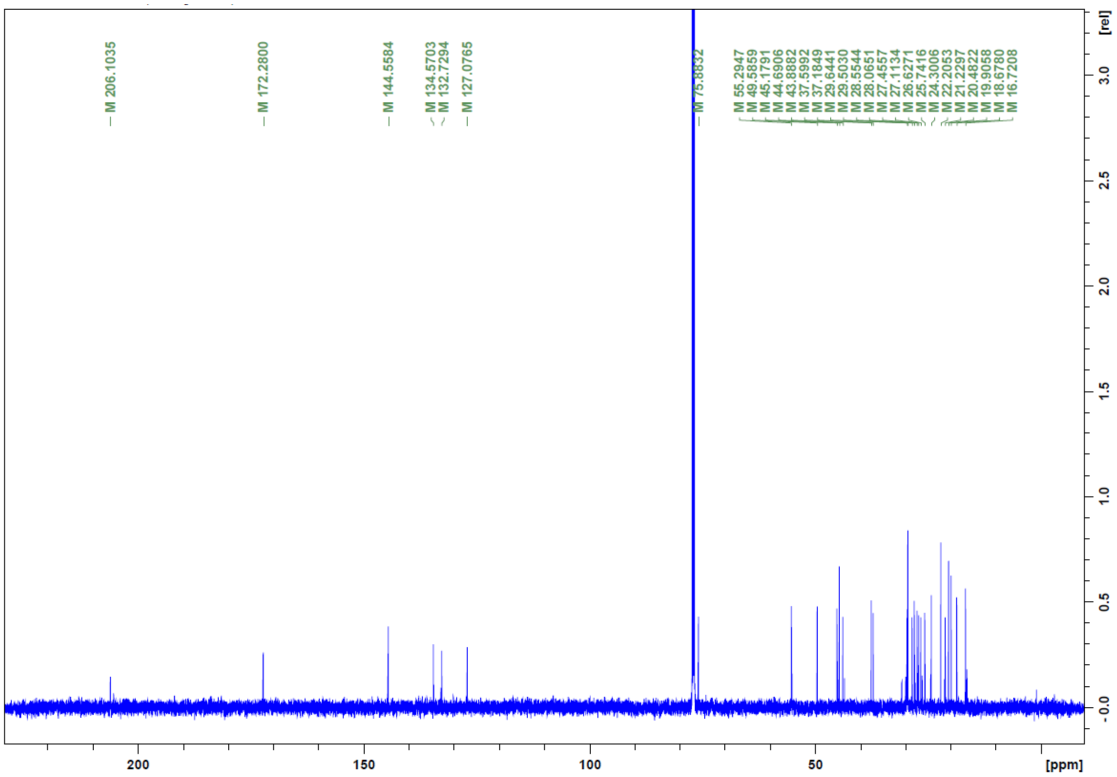

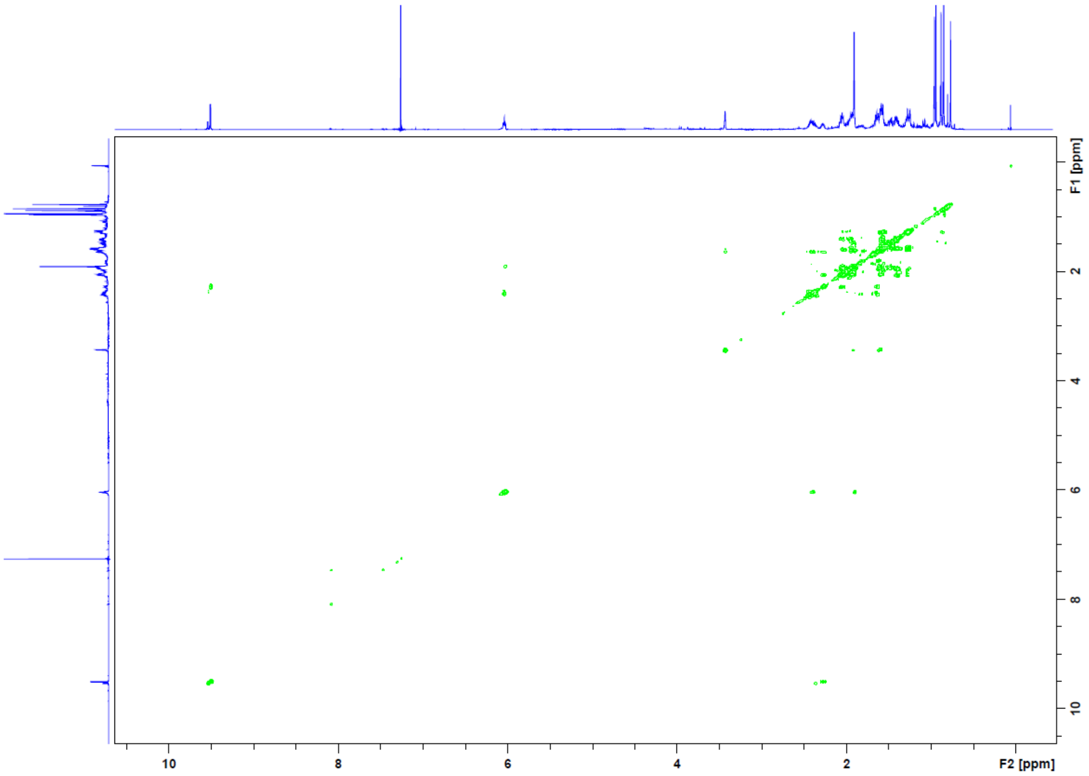
F1 [ppm]
2
4
6
8
10
10
8
6
4
2
F2 [ppm]

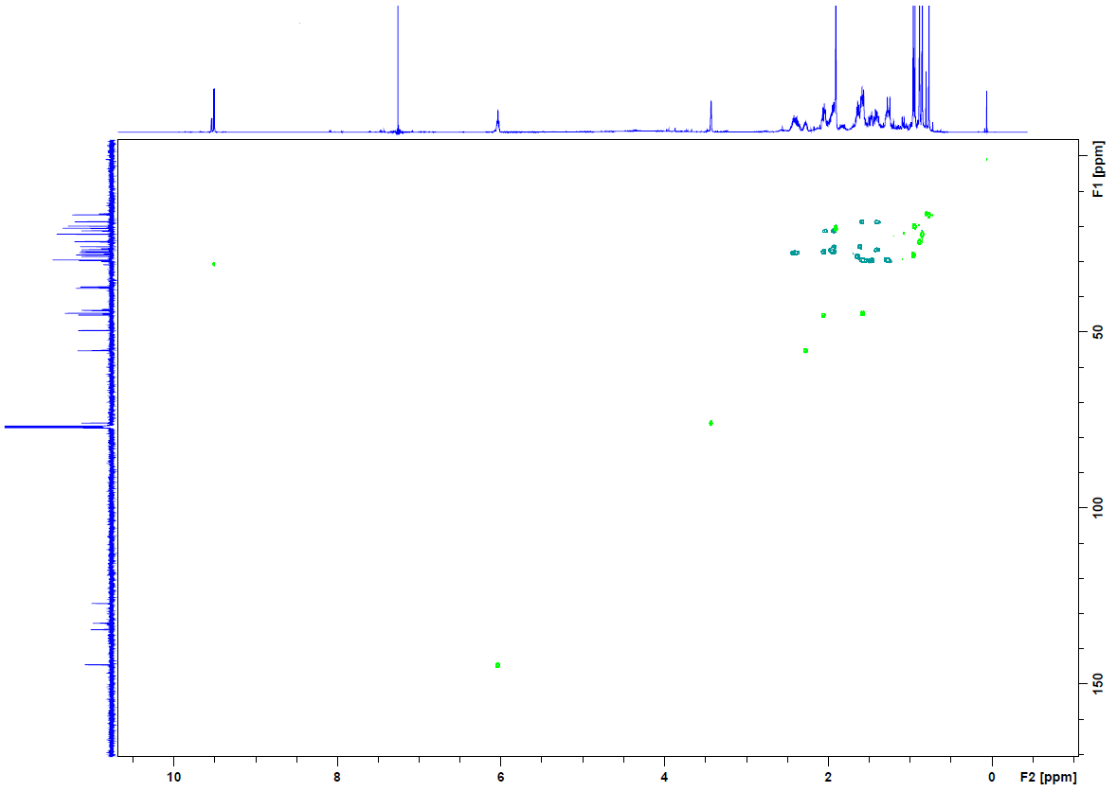
F1 [ppm]
50
100
150
10
8
6
4
2
0
F2 [ppm]

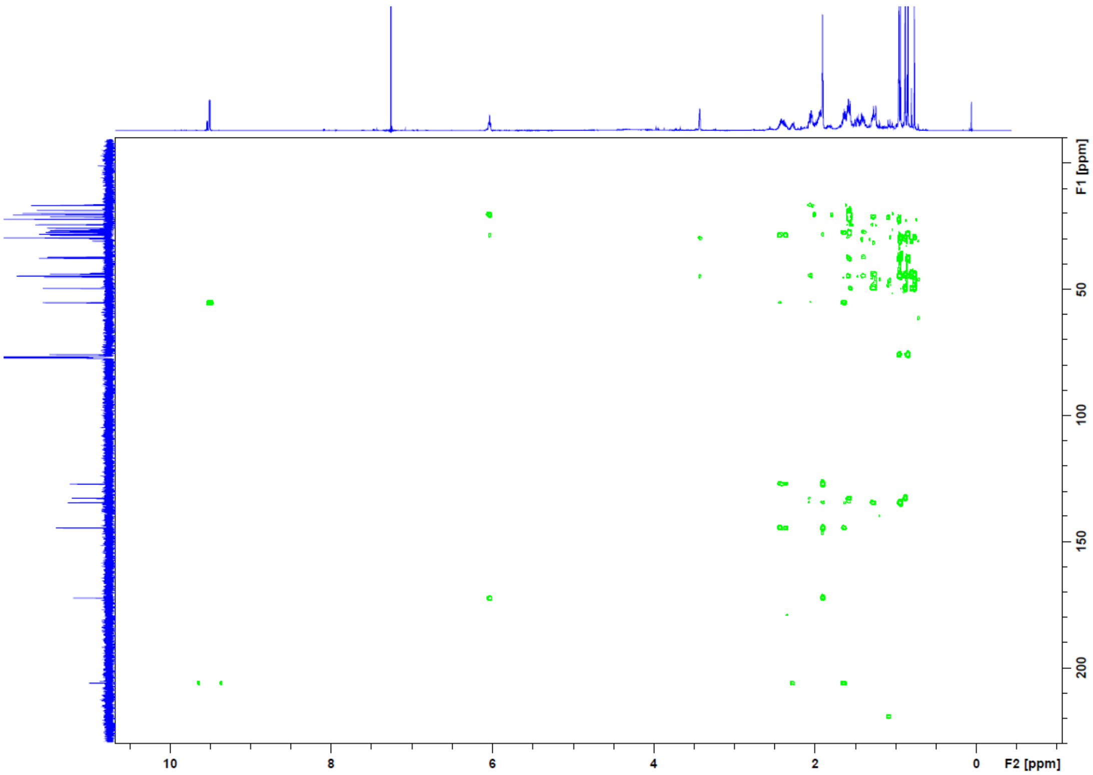

F1 [ppm]
50
100
150
200
10
8
6
4
2
0
F2 [ppm]

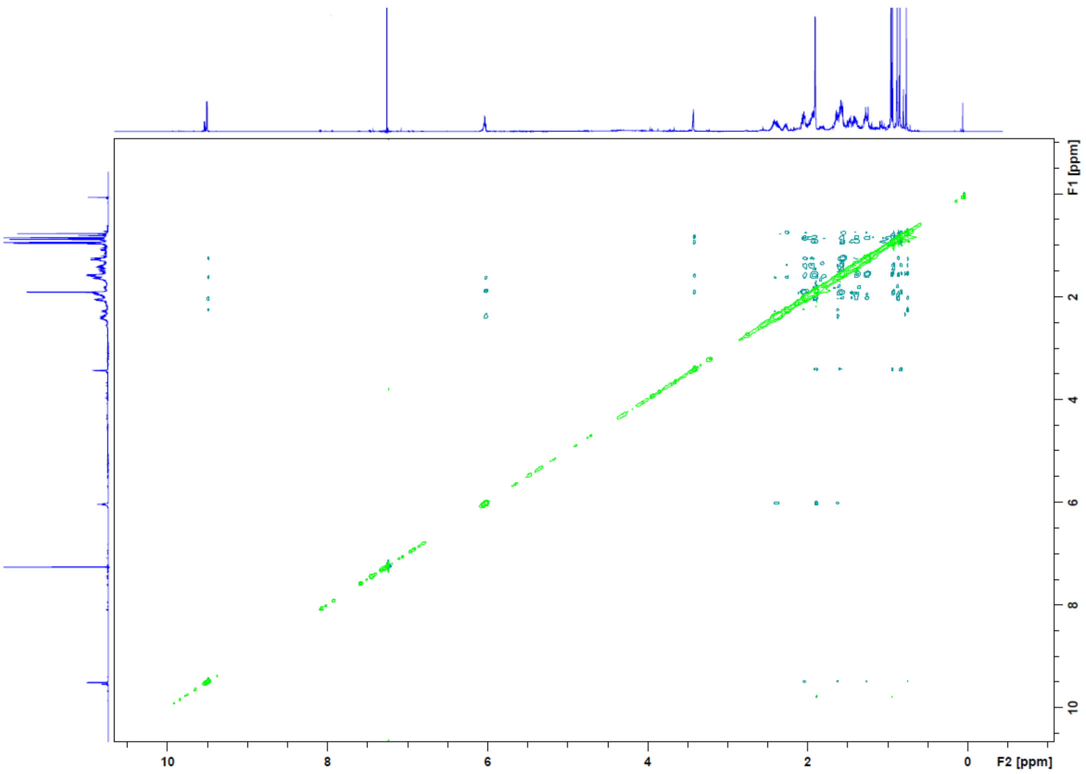

F1 [ppm]
2
4
6
8
10
10
8
6
4
2
0
F2 [ppm]

Figure S4. HRESIMS spectrum of **3**.

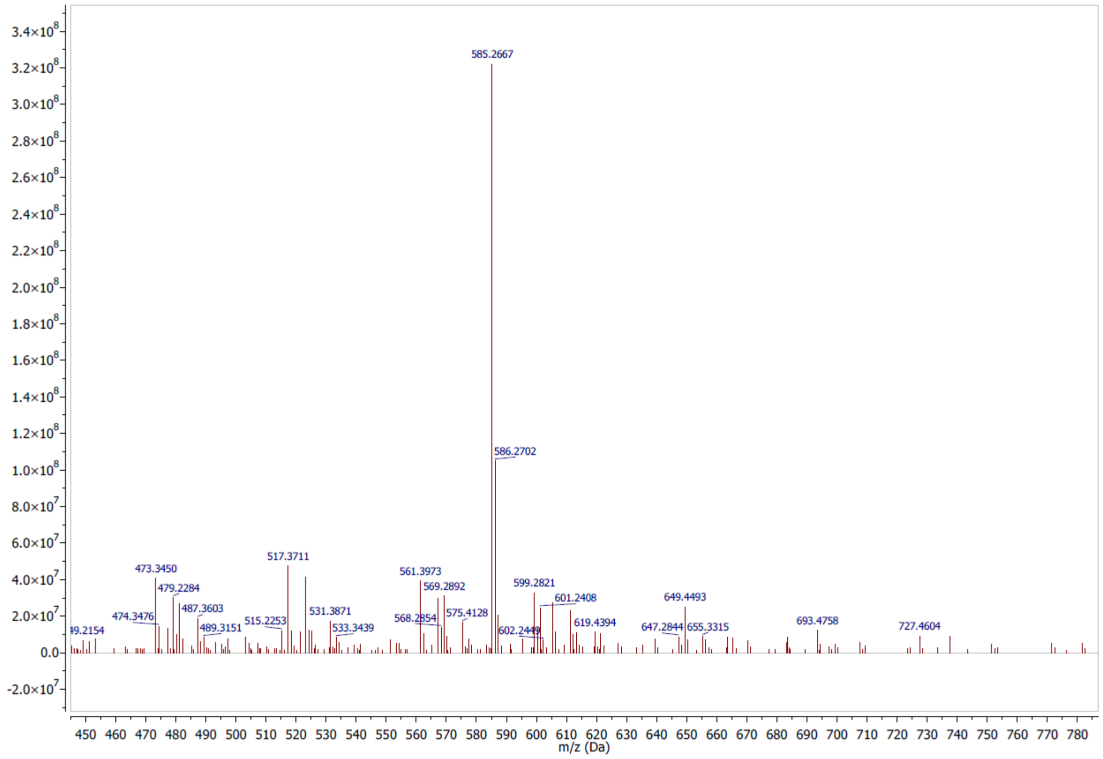

Figure S5. ^{1}H NMR, ^{1}H - ^{1}H COSY, HSQC, HMBC and ROESY spectra of **3** in $CDCl_3$.

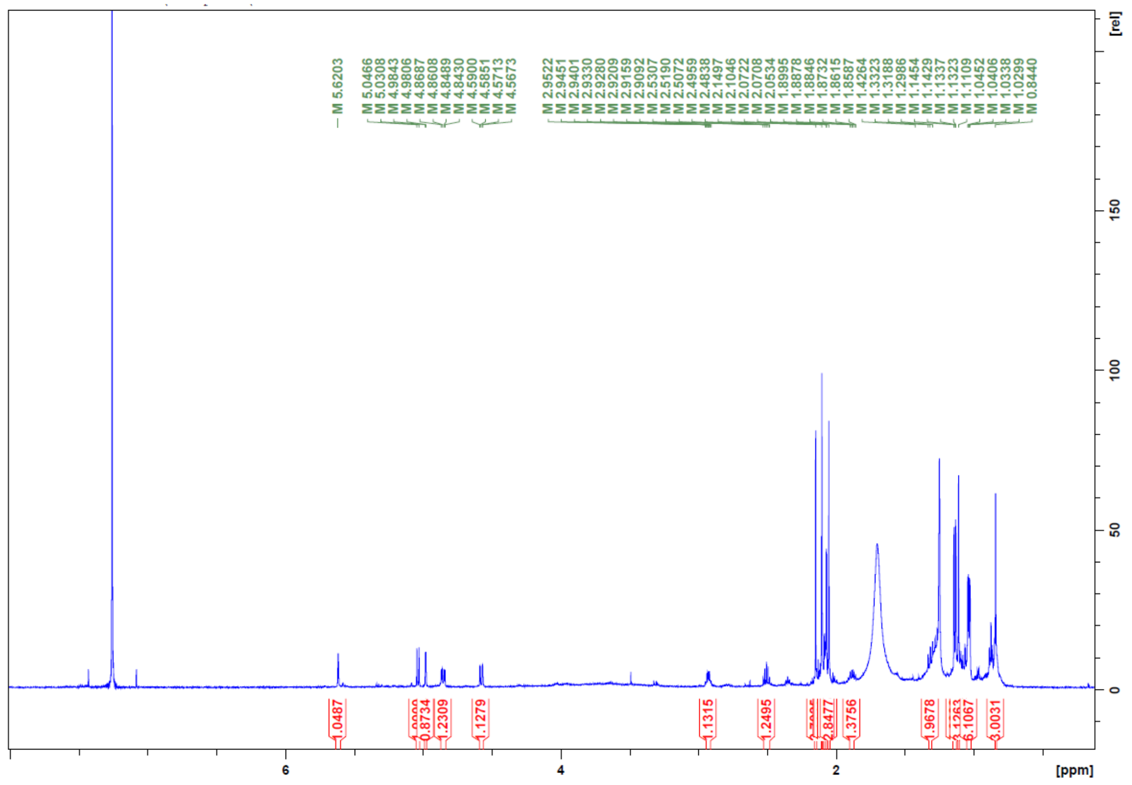

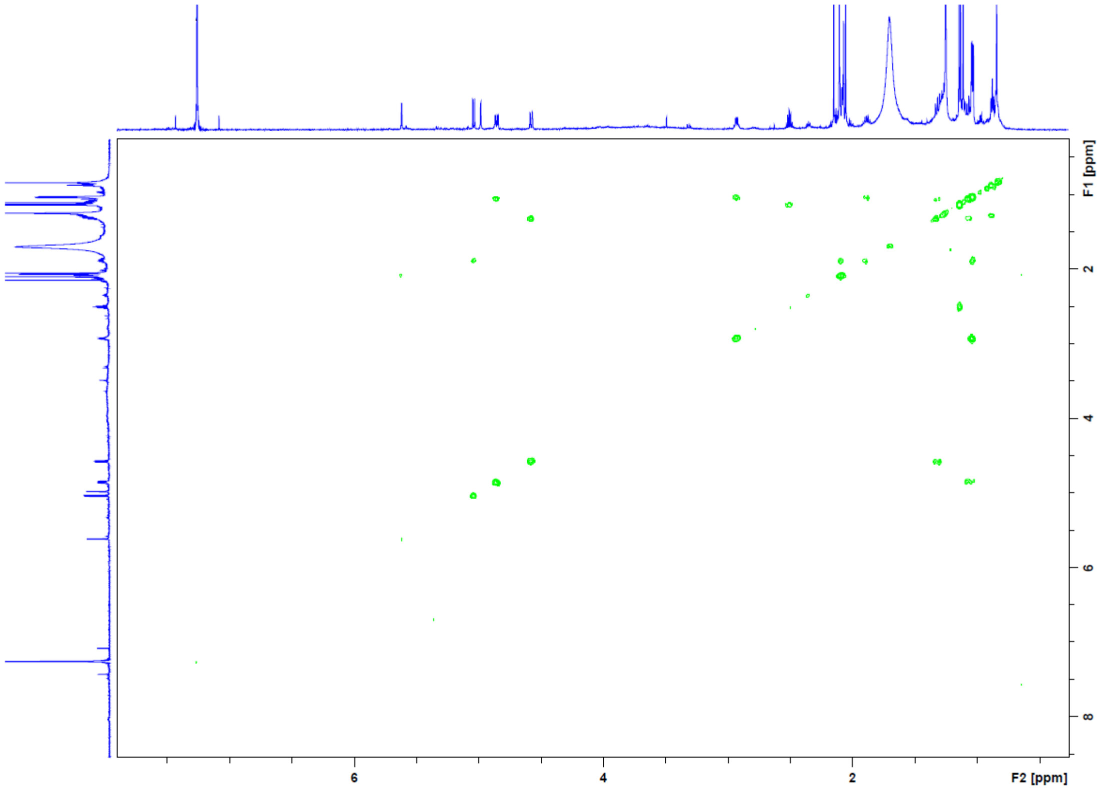

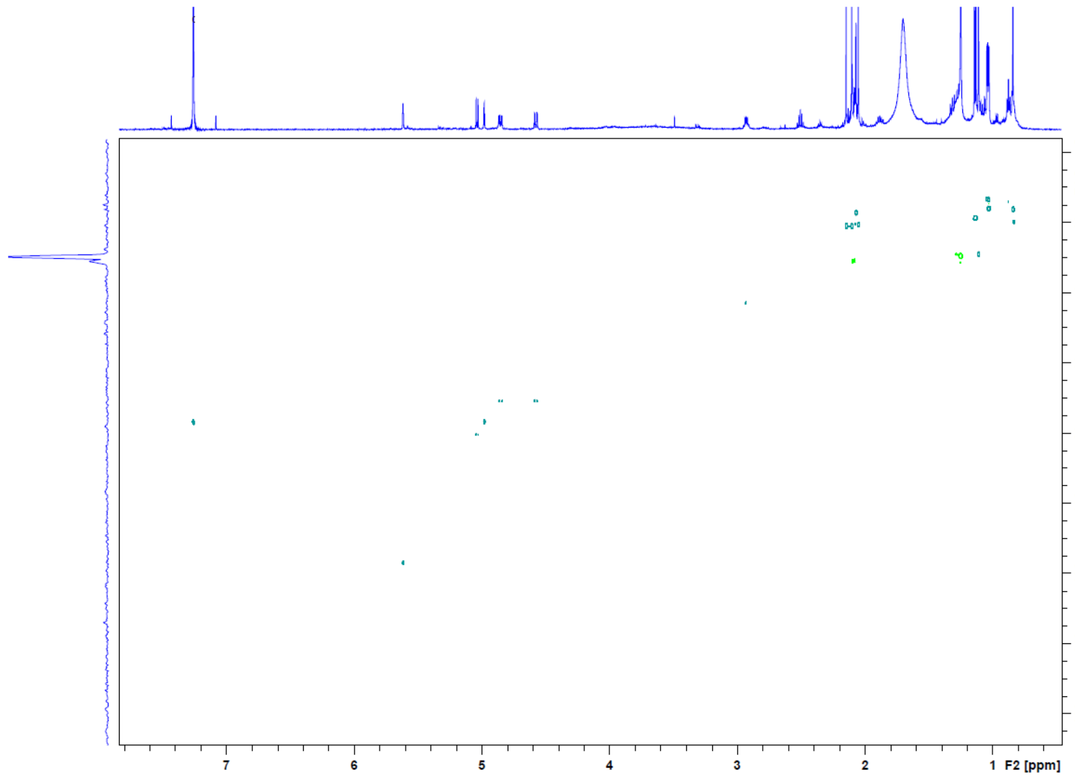
F1 [ppm]
20
40
60
80
100
120
140
160
7
6
5
4
3
2
1 F2 [ppm]

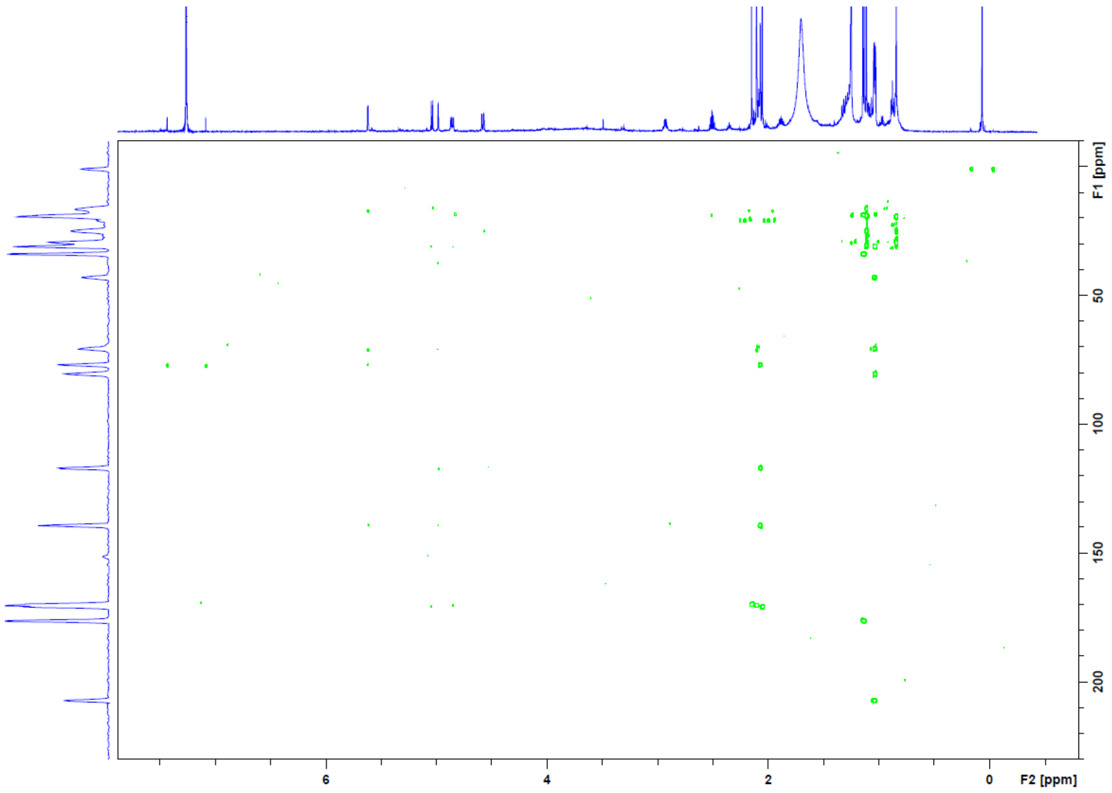
F1 [ppm]
50
100
150
200
6
4
2
0
F2 [ppm]

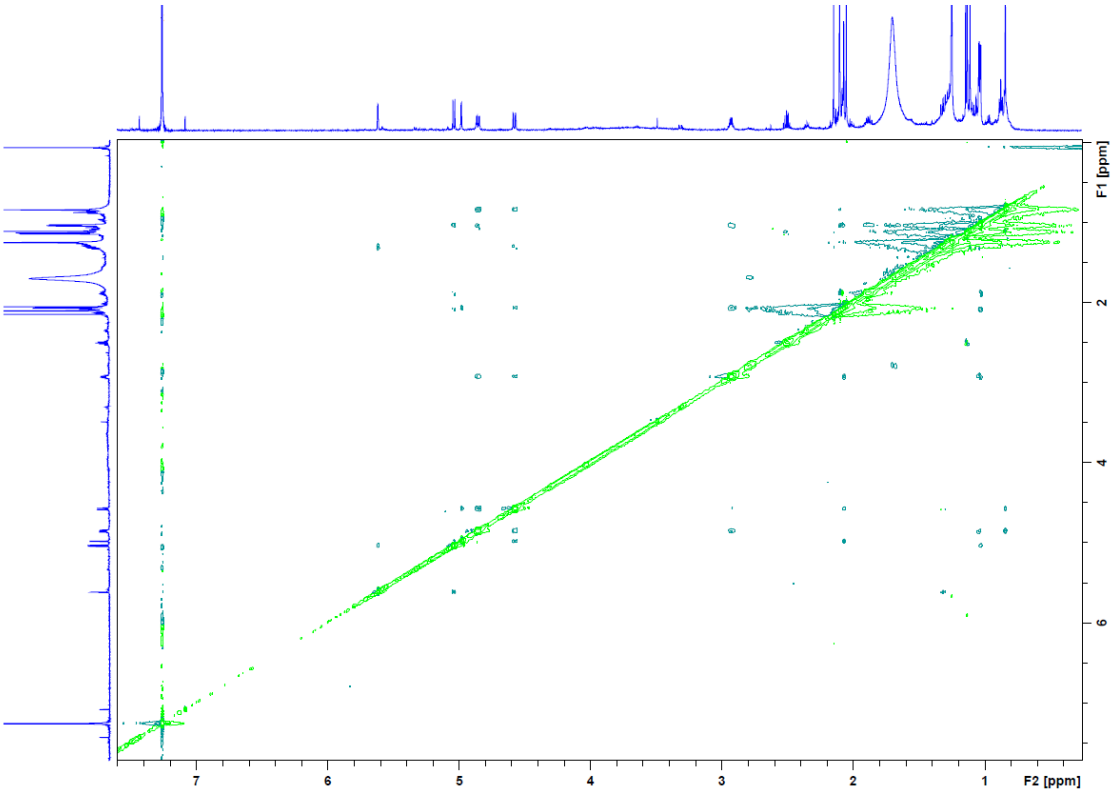
F1 [ppm]
2
4
6
7
6
5
4
3
2
1
F2 [ppm]

Figure S6. ^{1}H NMR, ^{13}C NMR, DEPT, ^{1}H - ^{1}H COSY, HSQC, HMBC and ROESY spectra of **4** in $CDCl_3$.

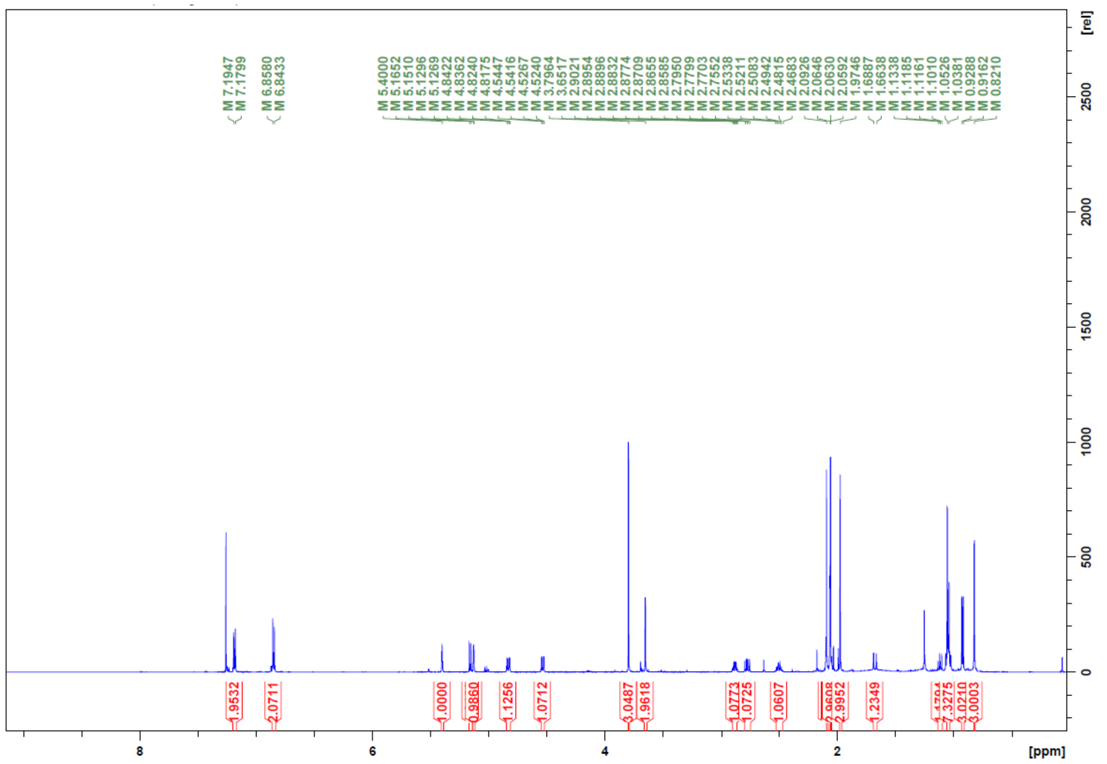

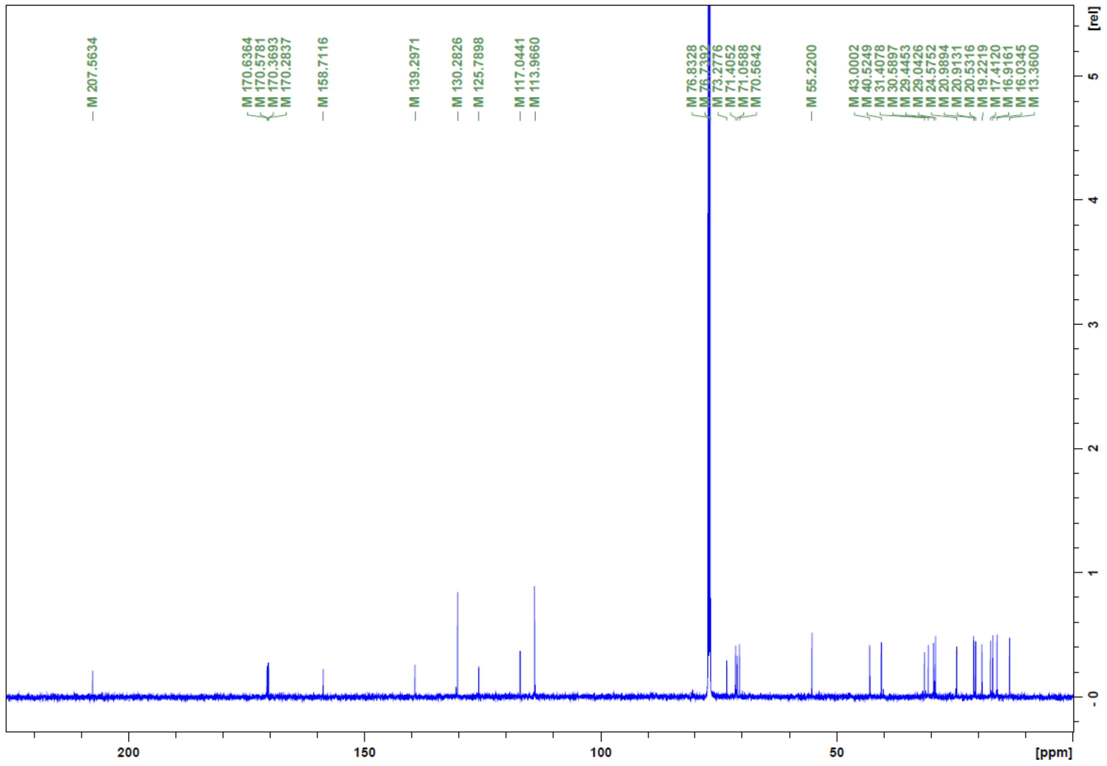

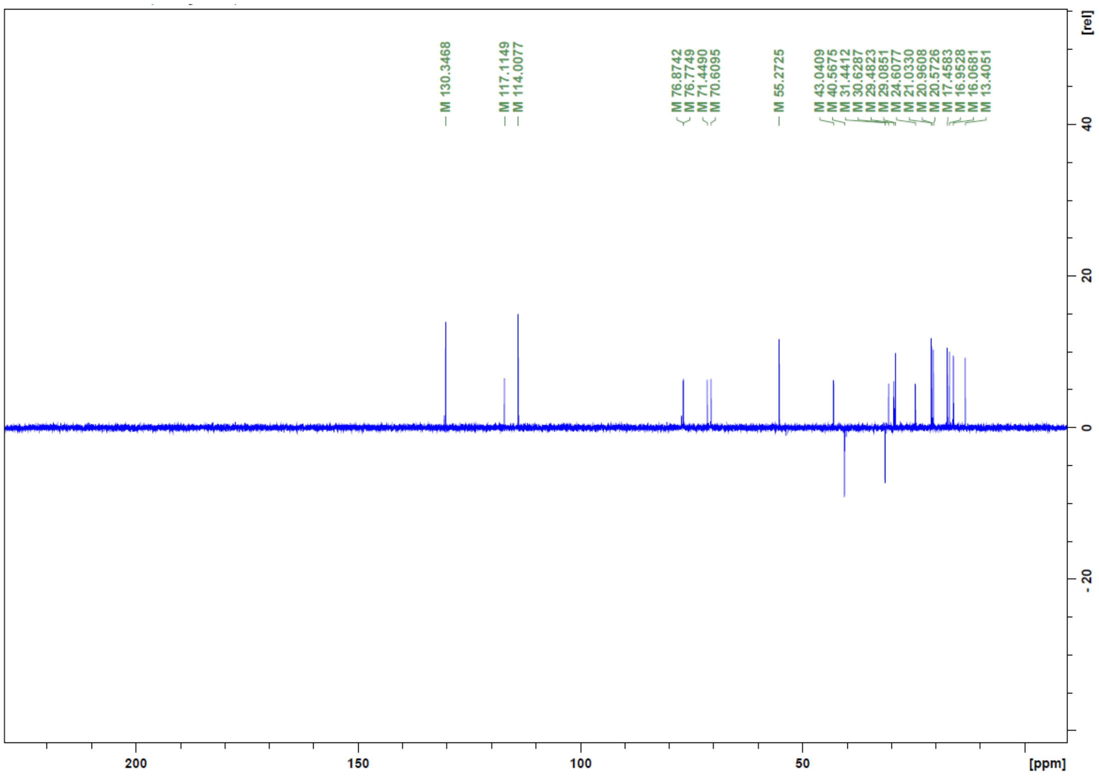
M 130.3468
M 117.1149
M 114.0077
M 76.8742
M 76.7749
M 71.4490
M 70.6095
M 55.2725
M 43.0409
M 40.5675
M 31.4412
M 30.6287
M 29.4823
M 29.0851
M 24.6077
M 21.0330
M 20.9608
M 20.5726
M 17.4583
M 16.9528
M 16.0681
M 13.4051
[rel]
40
20
0
-20
200
150
100
50
[ppm]

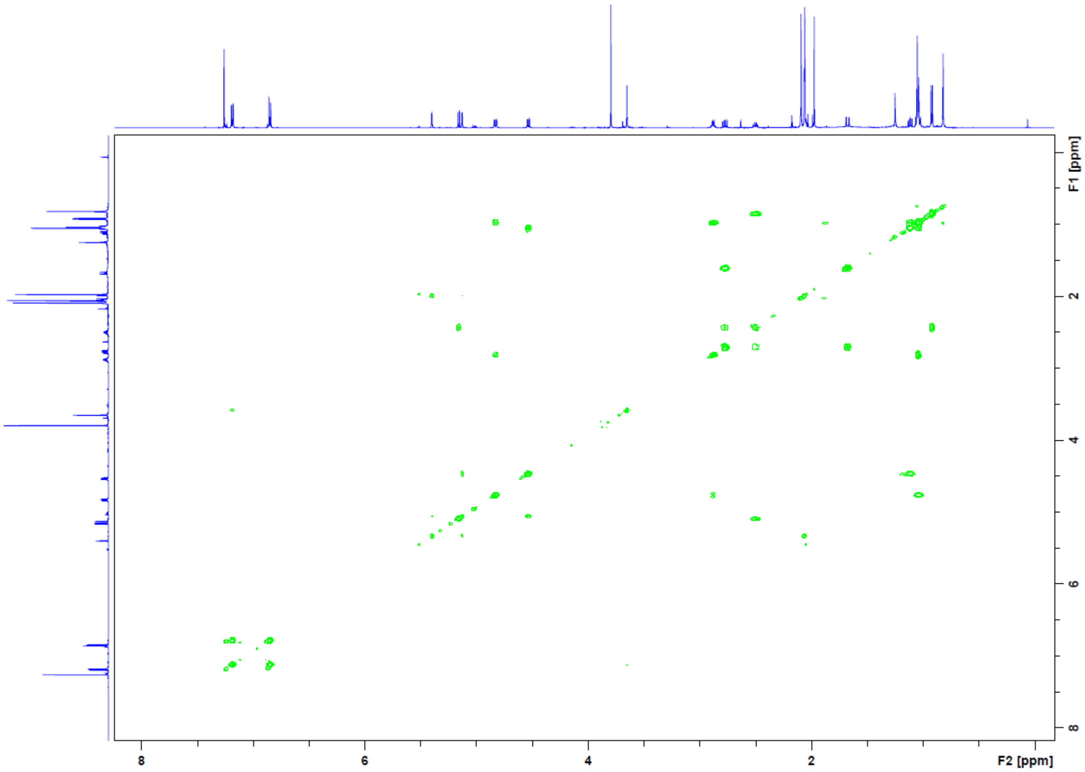
F1 [ppm]
2
4
6
8
8
6
4
2
F2 [ppm]

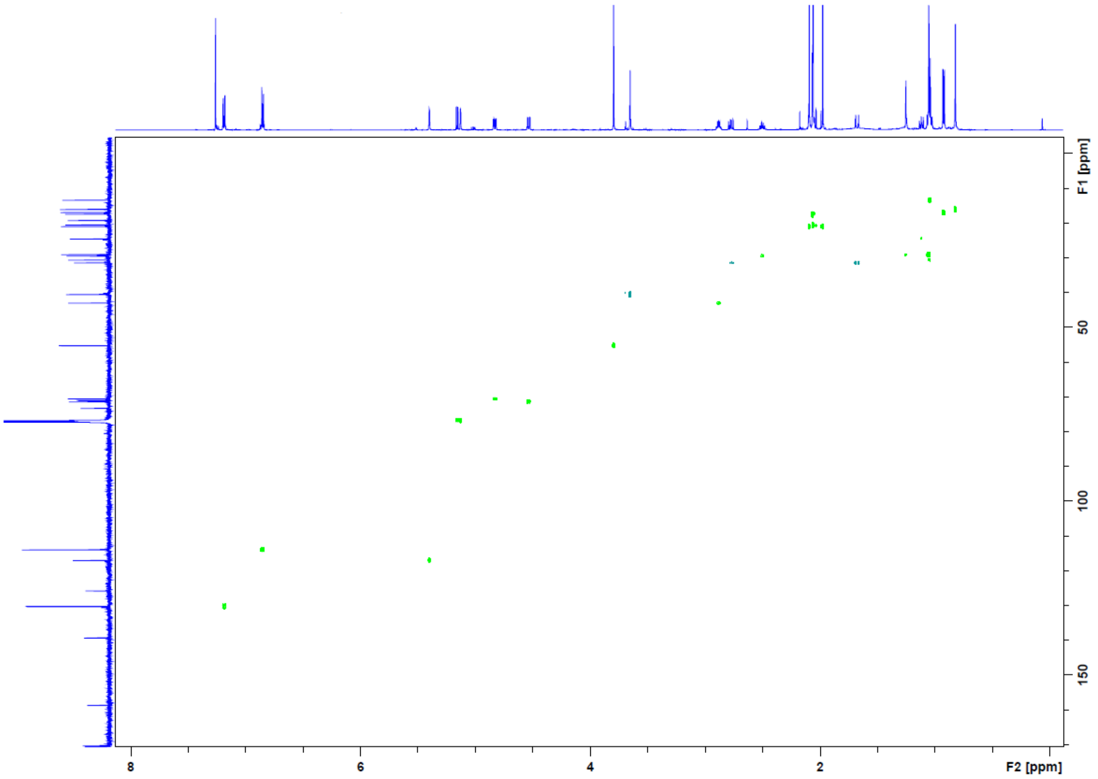
F1 [ppm]
50
100
150
8
6
4
2
F2 [ppm]

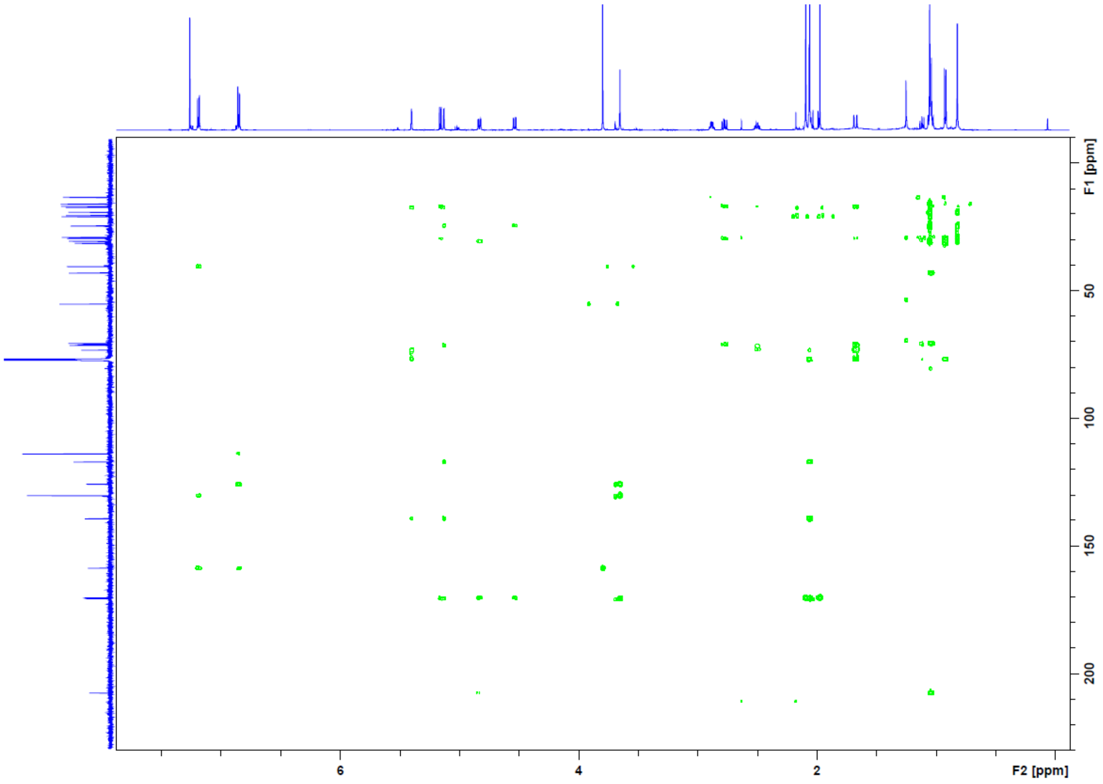
F1 [ppm]
50
100
150
200
6
4
2
F2 [ppm]

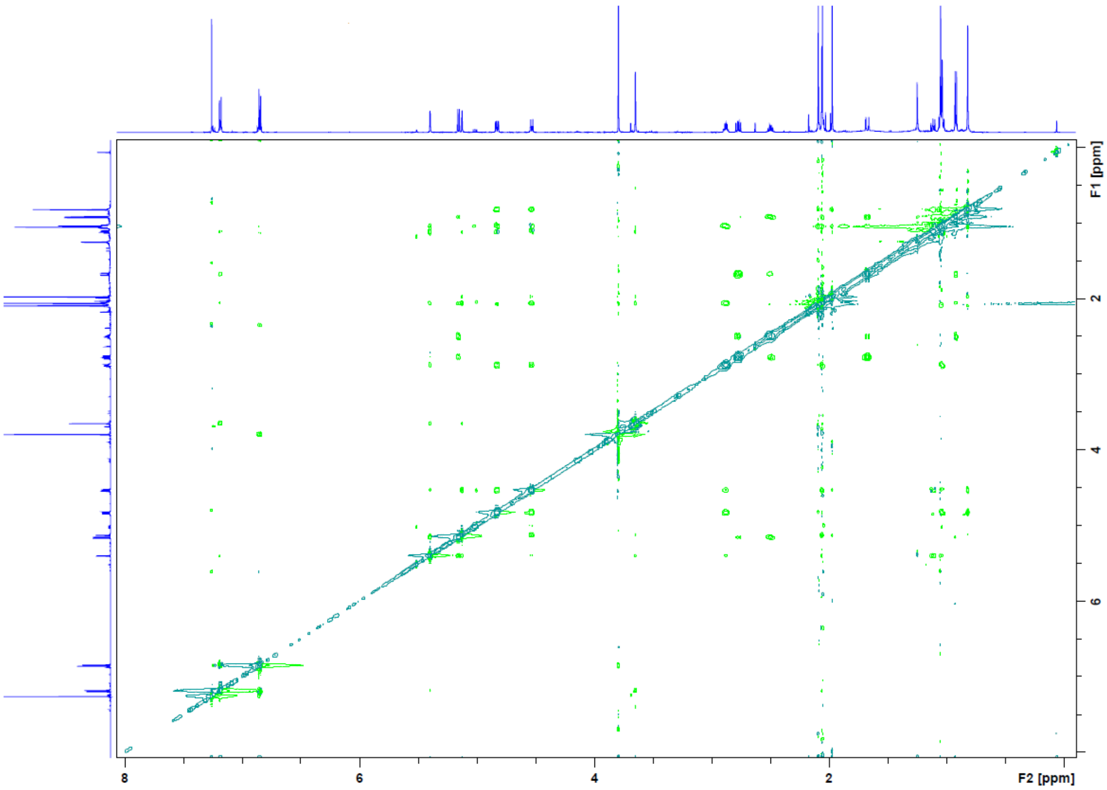
F1 [ppm]
2
4
6
8
6
4
2
F2 [ppm]

Figure S7. ^{1}H NMR, ^{13}C NMR, HSQC and HMBC spectra of **4** in acetone-d_6.

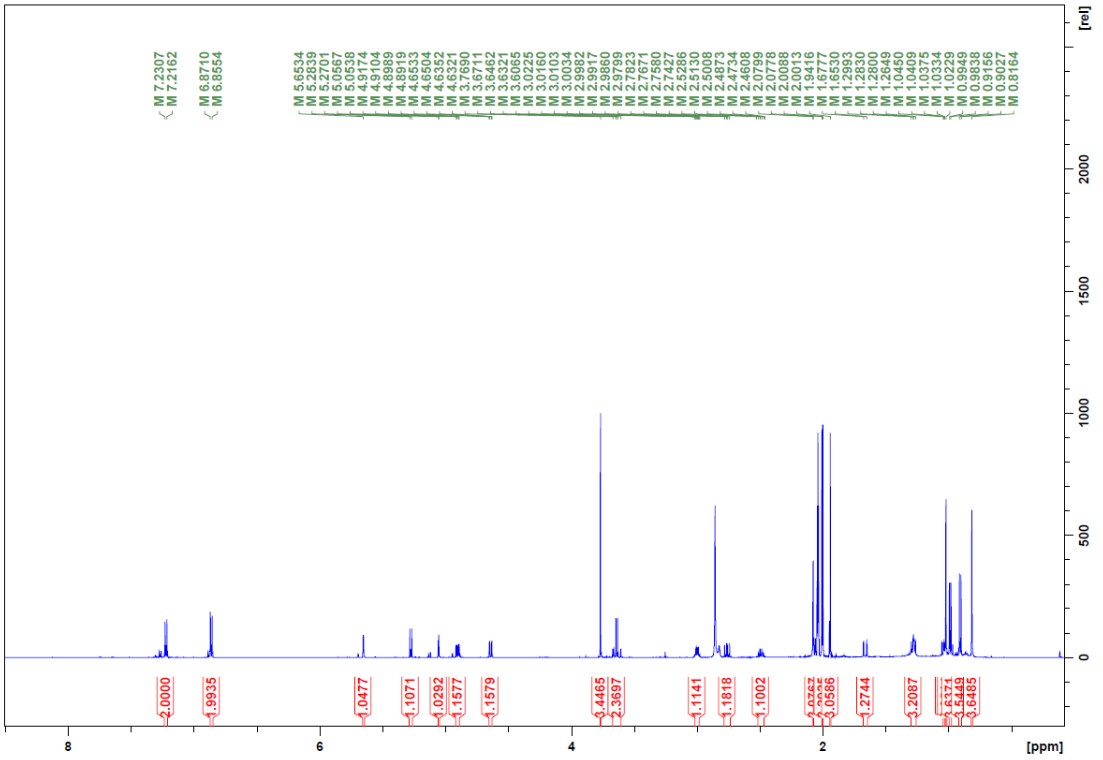

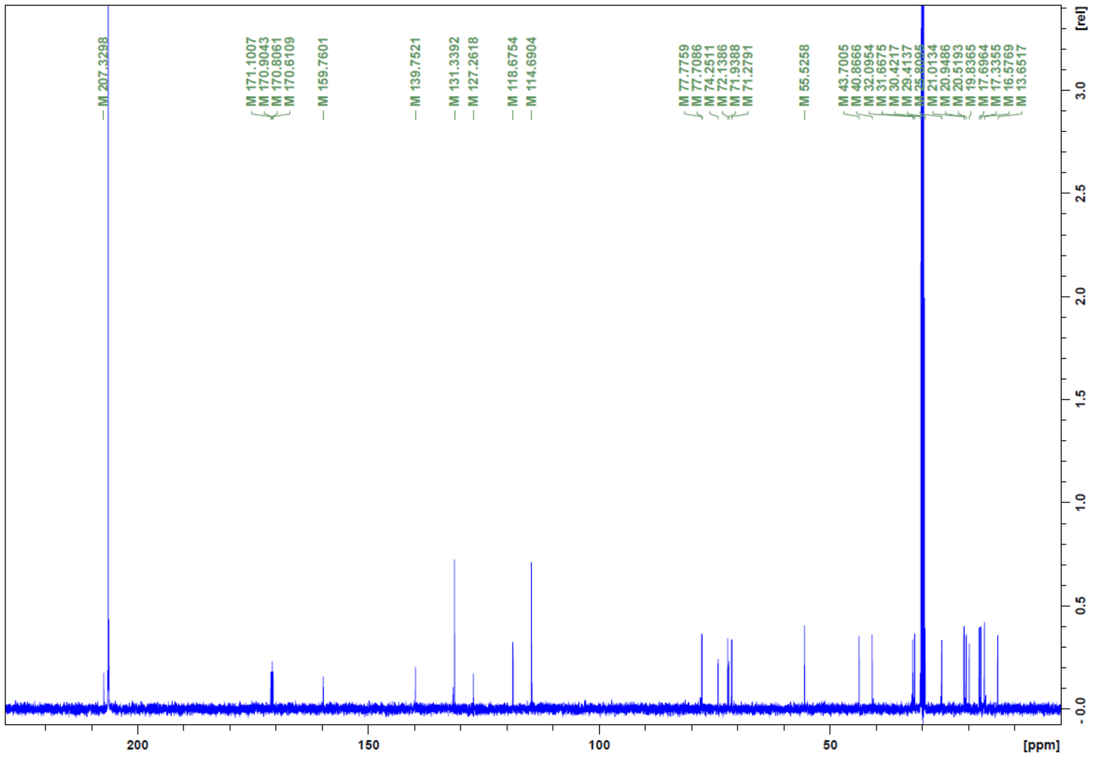

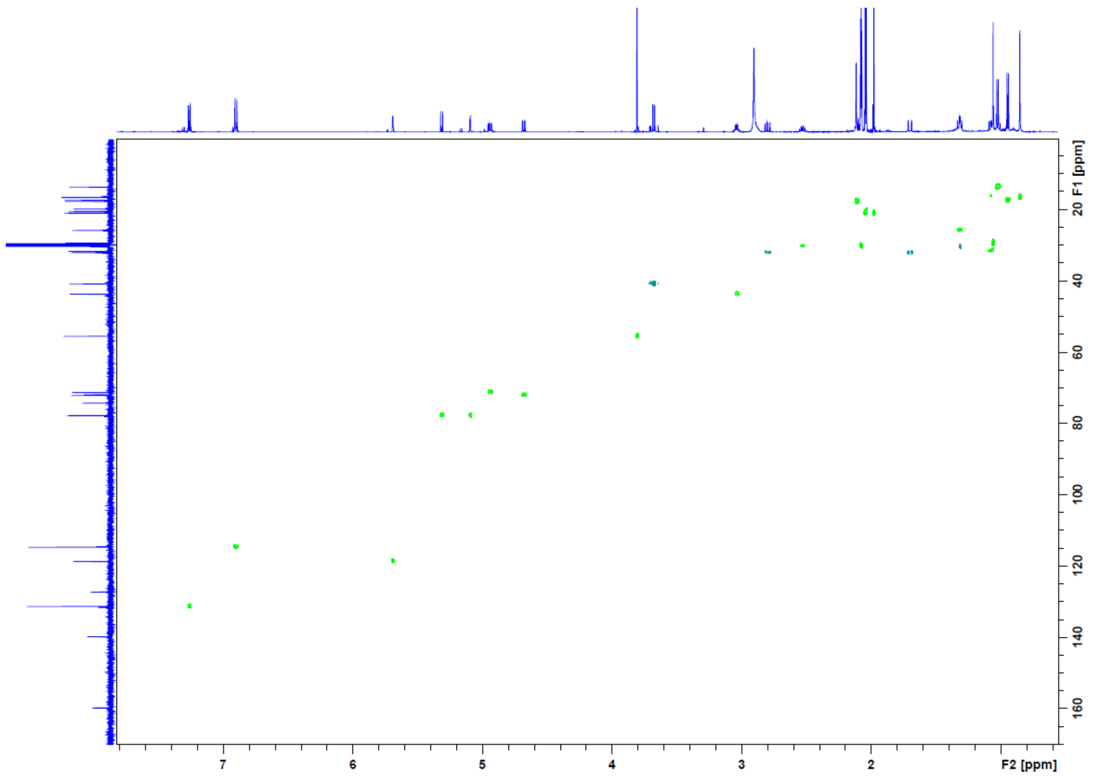
F1 [ppm]
F2 [ppm]

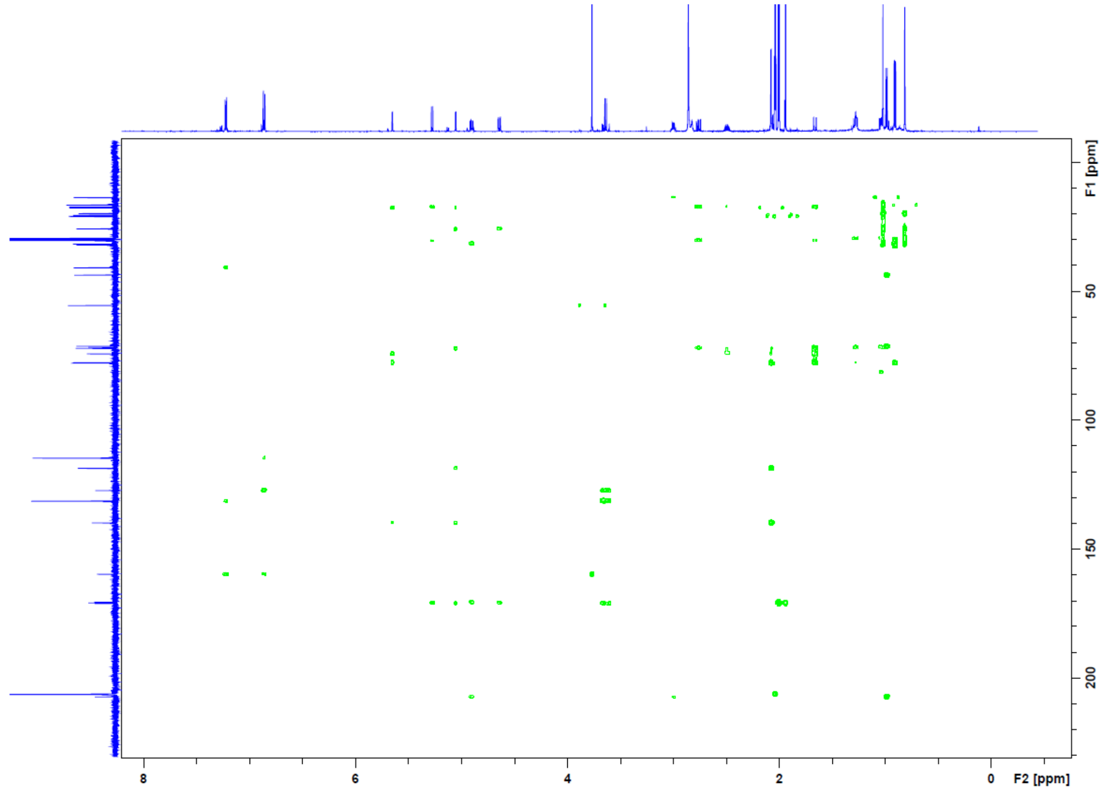
F1 [ppm]
F2 [ppm]

Figure S8. ^{1}H NMR, ^{13}C NMR, HSQC, HMBC and ROESY spectra of **5** in $CDCl_3$.

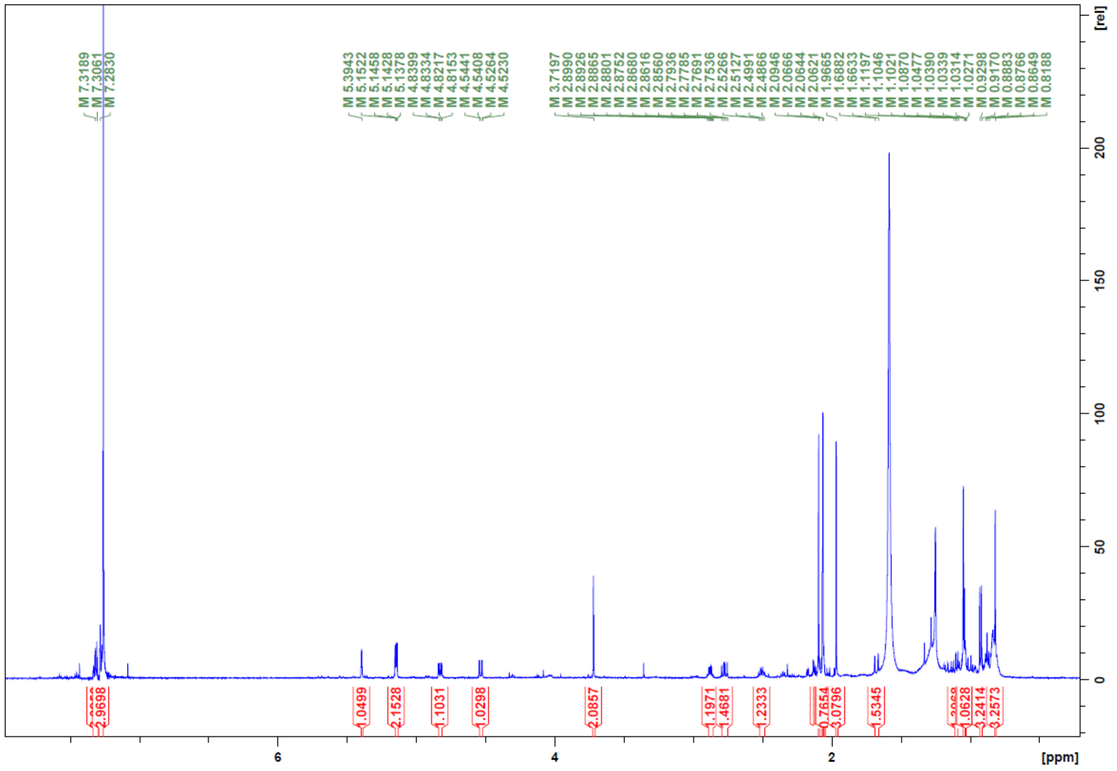

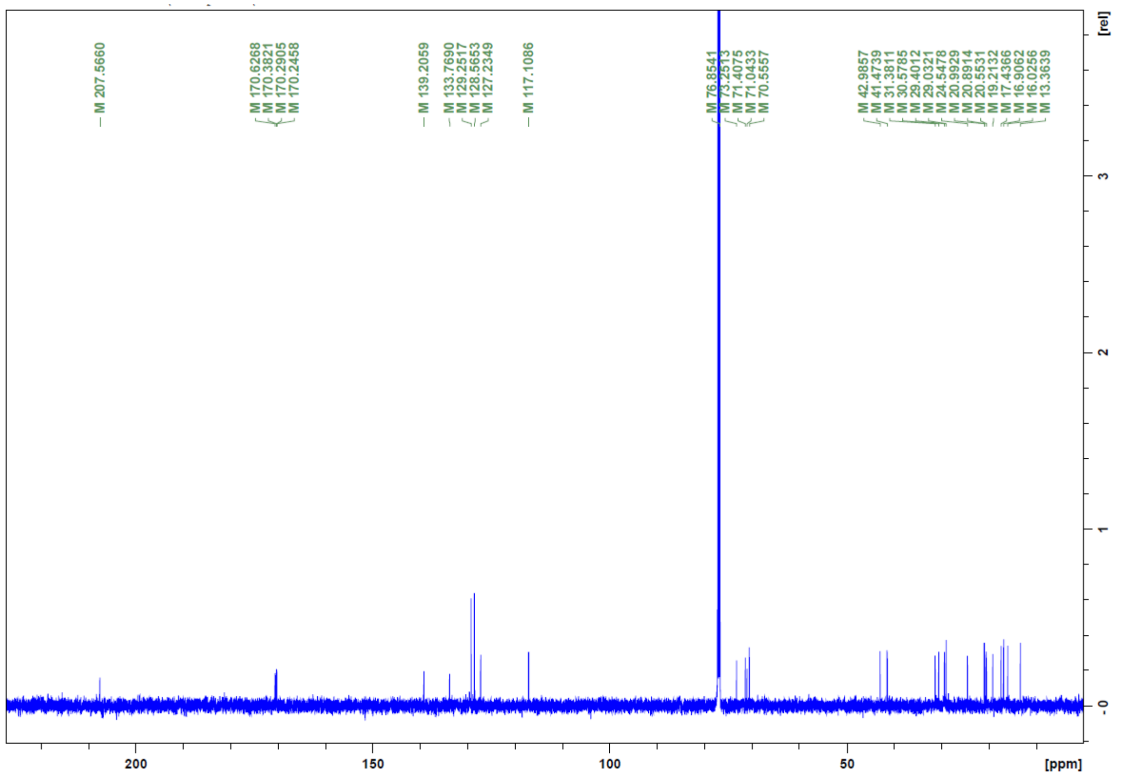

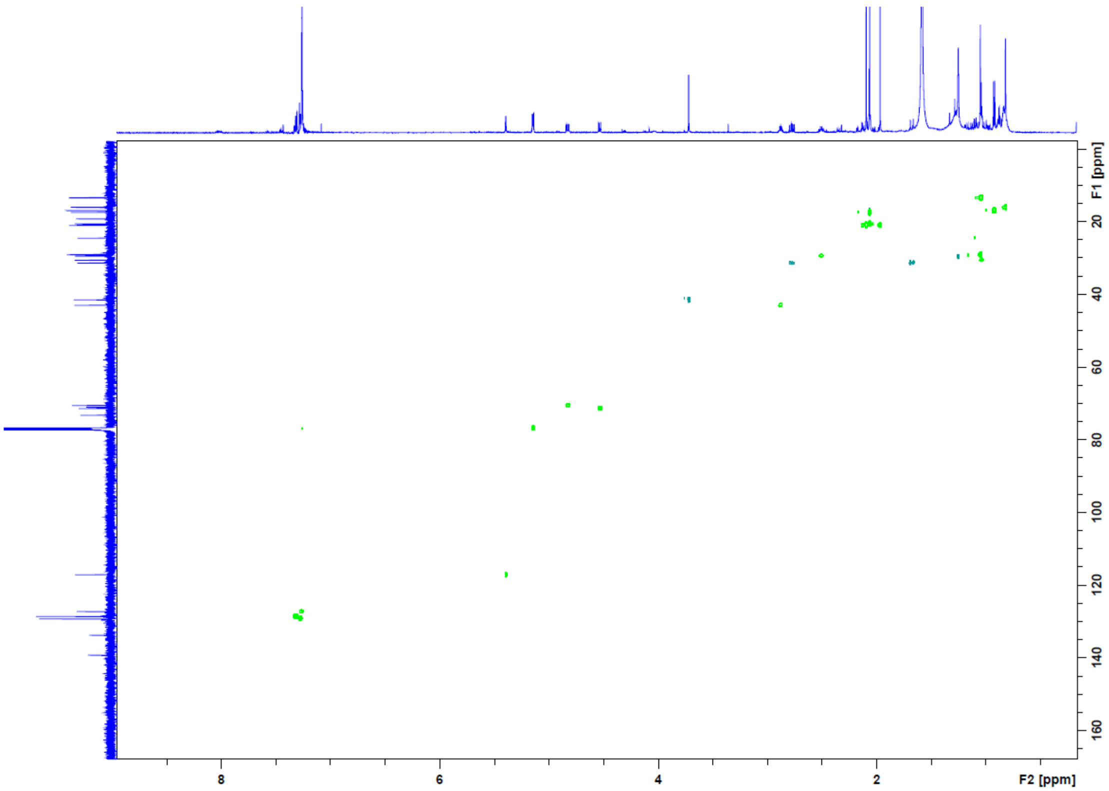
F1 [ppm]
F2 [ppm]

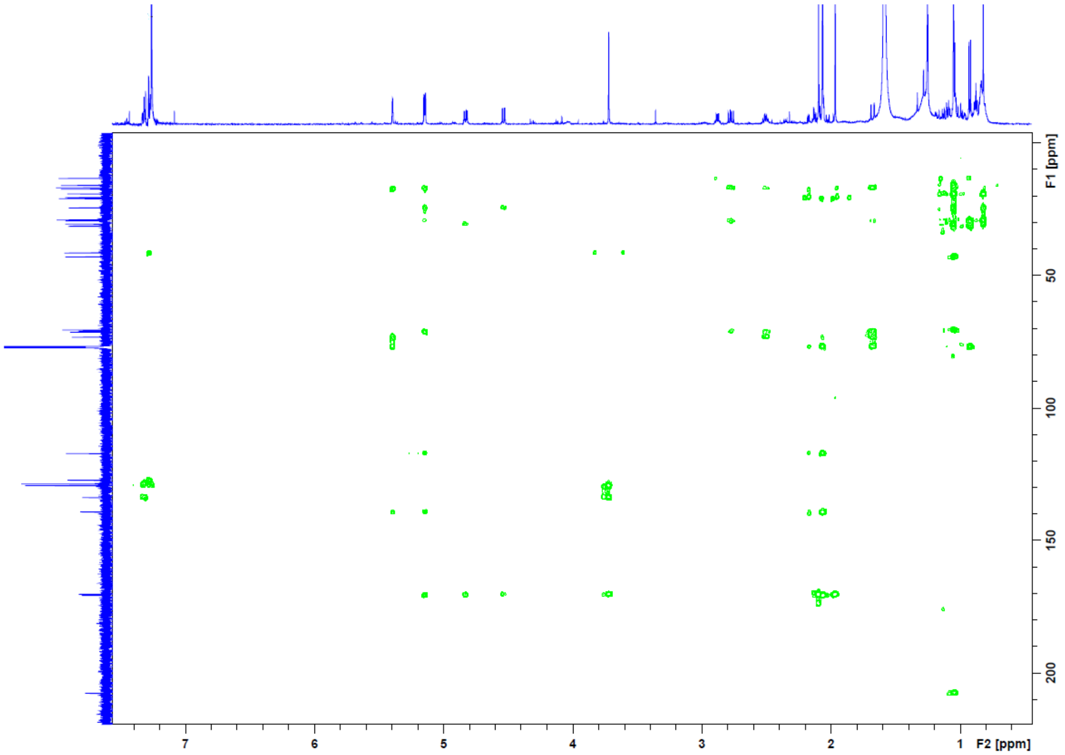
F1 [ppm]
F2 [ppm]

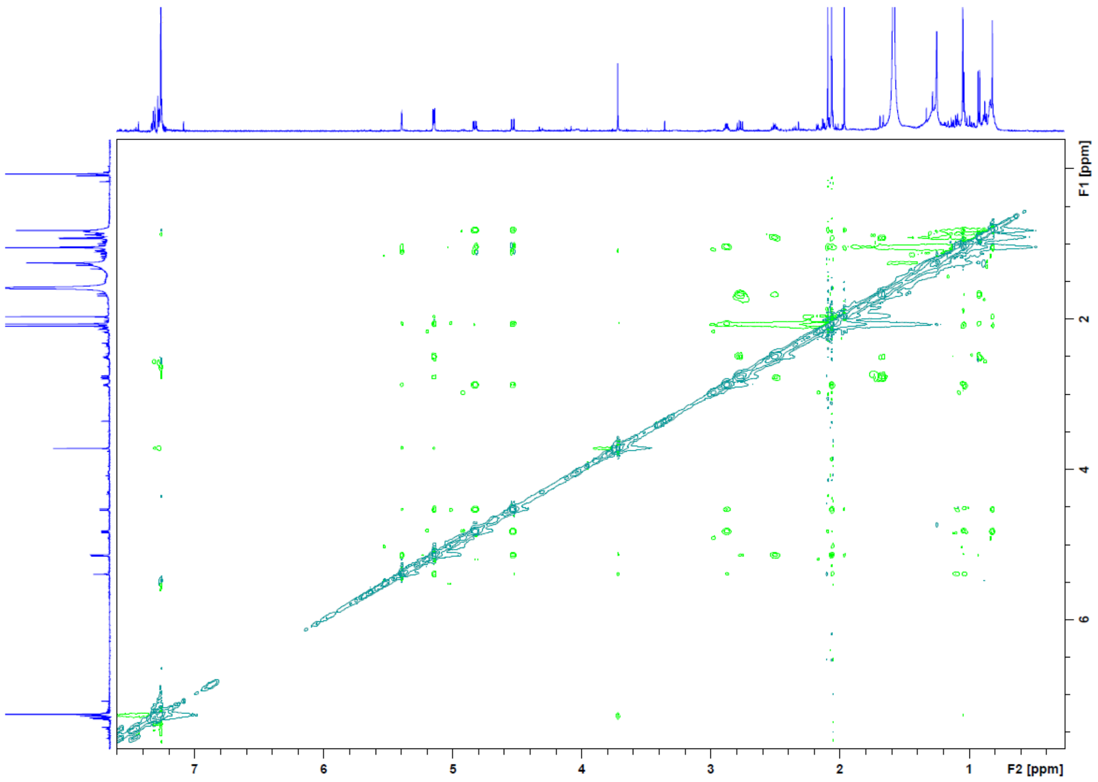
F1 [ppm]
2
4
6
7
6
5
4
3
2
1
F2 [ppm]

Figure S9. ^{1}H NMR, ^{13}C NMR, HSQC, HMBC and ROESY spectra of **5** in acetone-d_6.

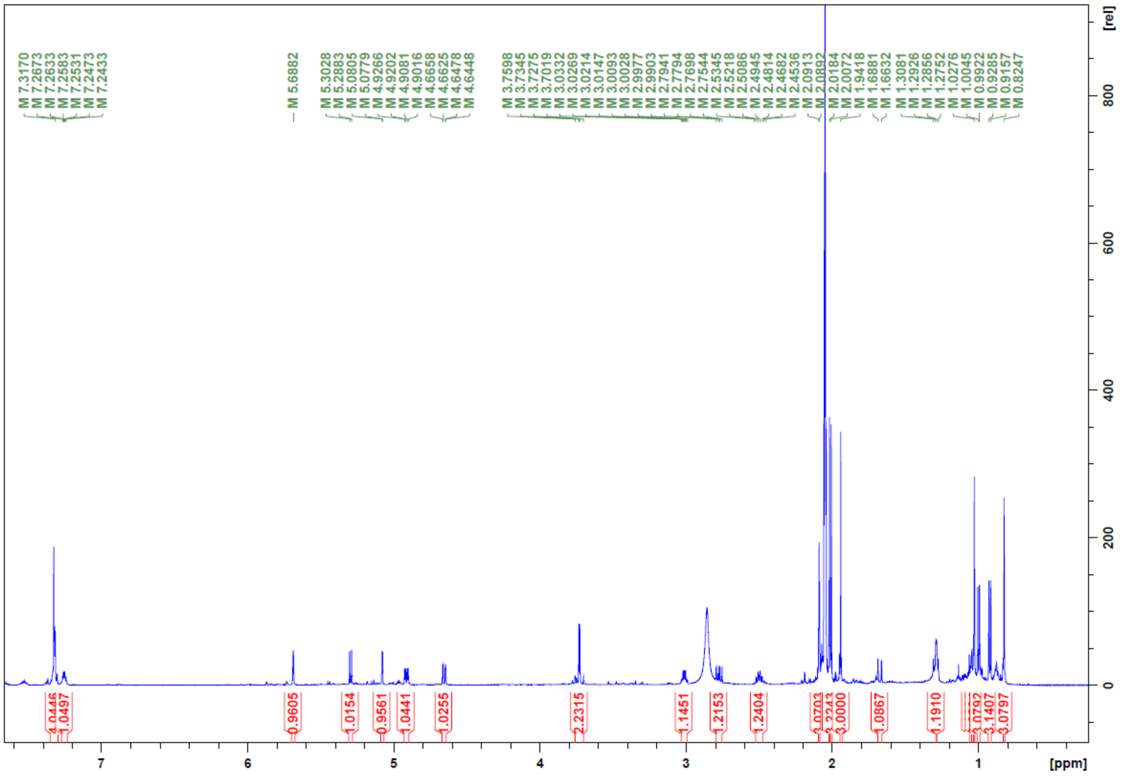

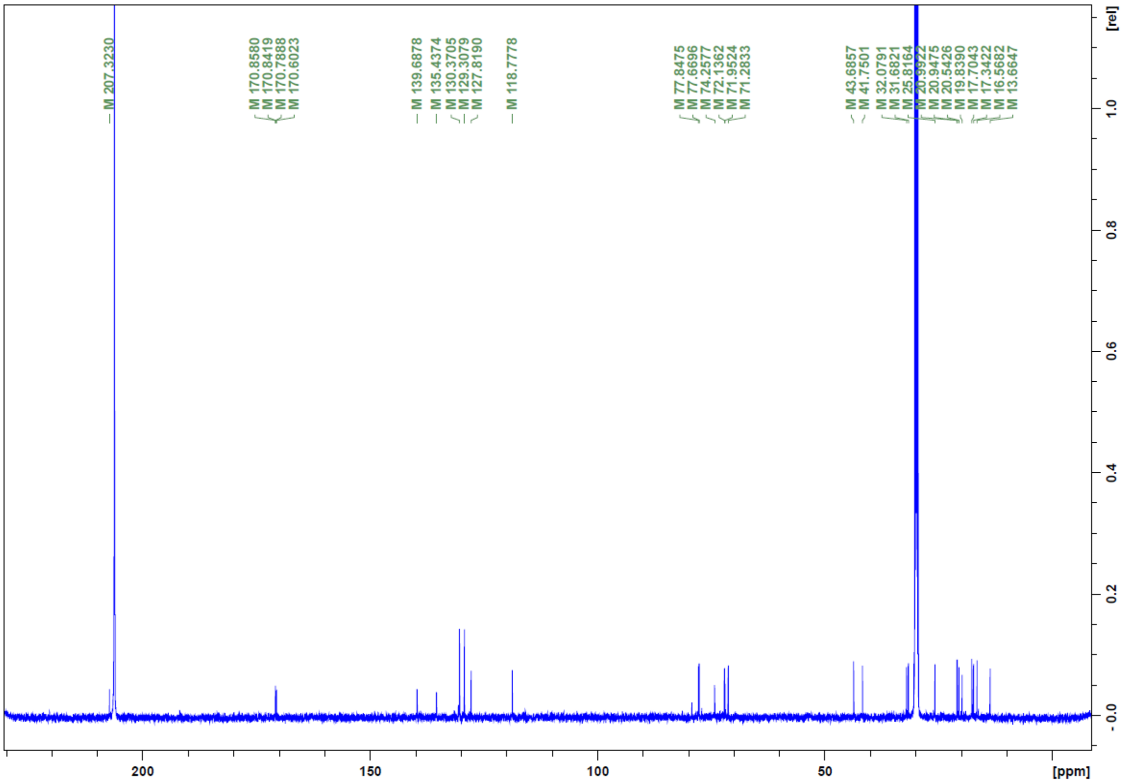

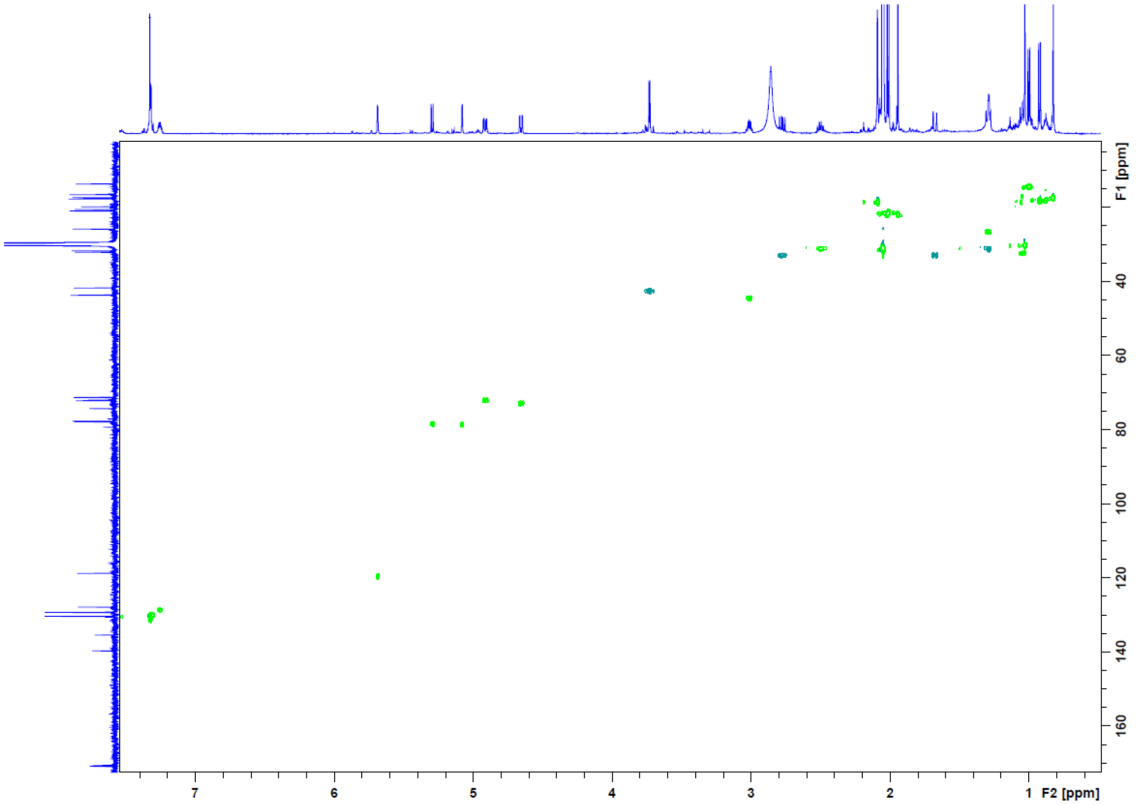
F1 [ppm]
20
40
60
80
100
120
140
160
7
6
5
4
3
2
1 F2 [ppm]

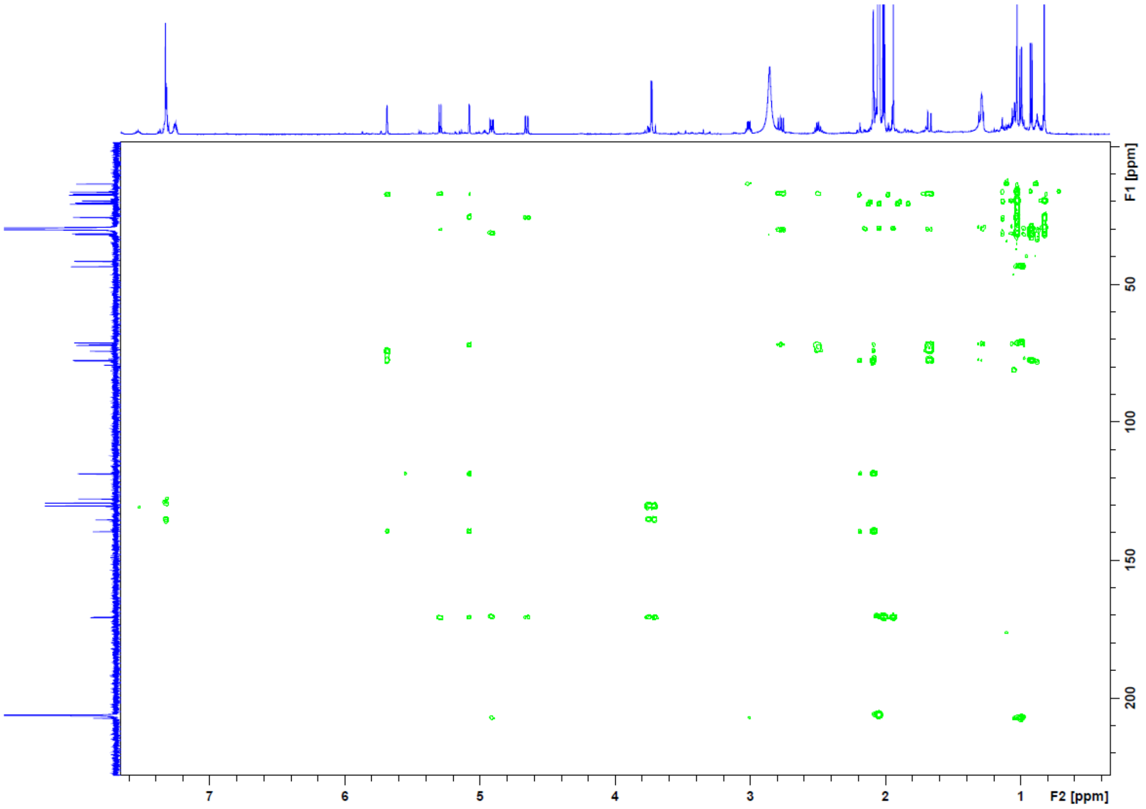
F1 [ppm]
50
100
150
200
7
6
5
4
3
2
1
F2 [ppm]

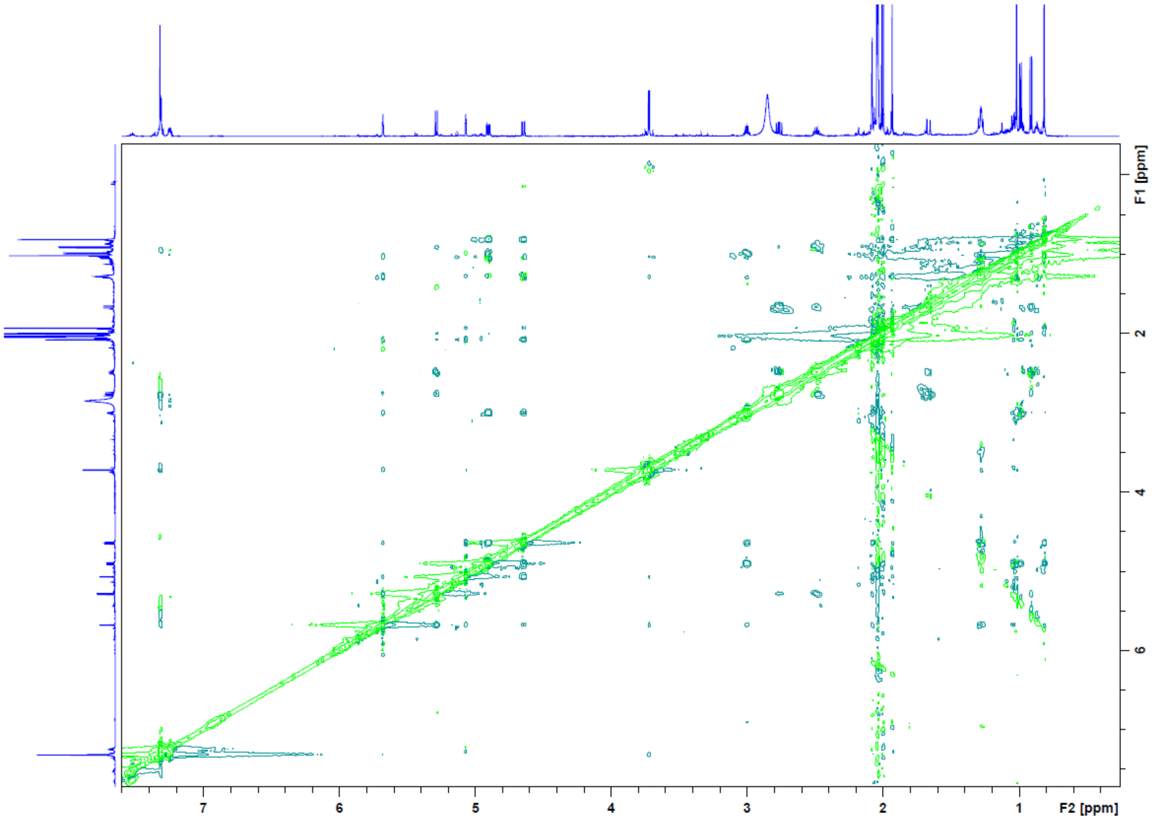
F1 [ppm]
F2 [ppm]

Figure S10. ^{1}H NMR spectrum of **6** in $CDCl_3$.

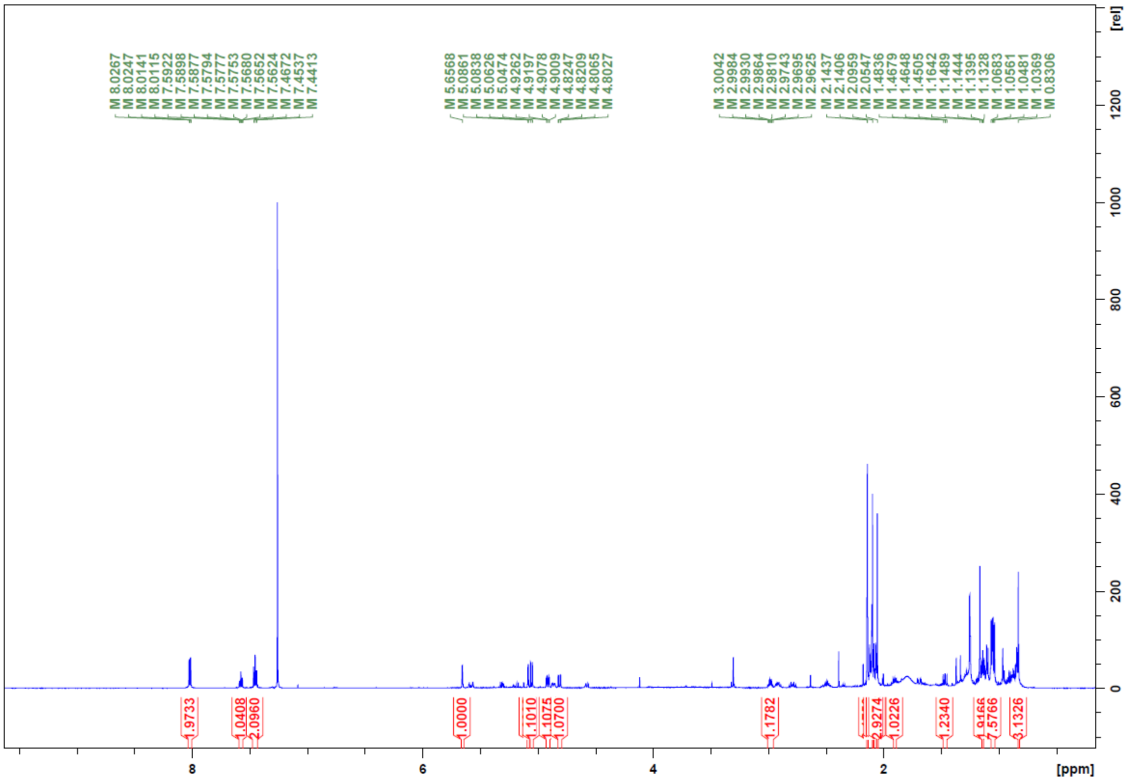

Figure S11. ^{1}H NMR spectrum of **7** in $CDCl_3$.

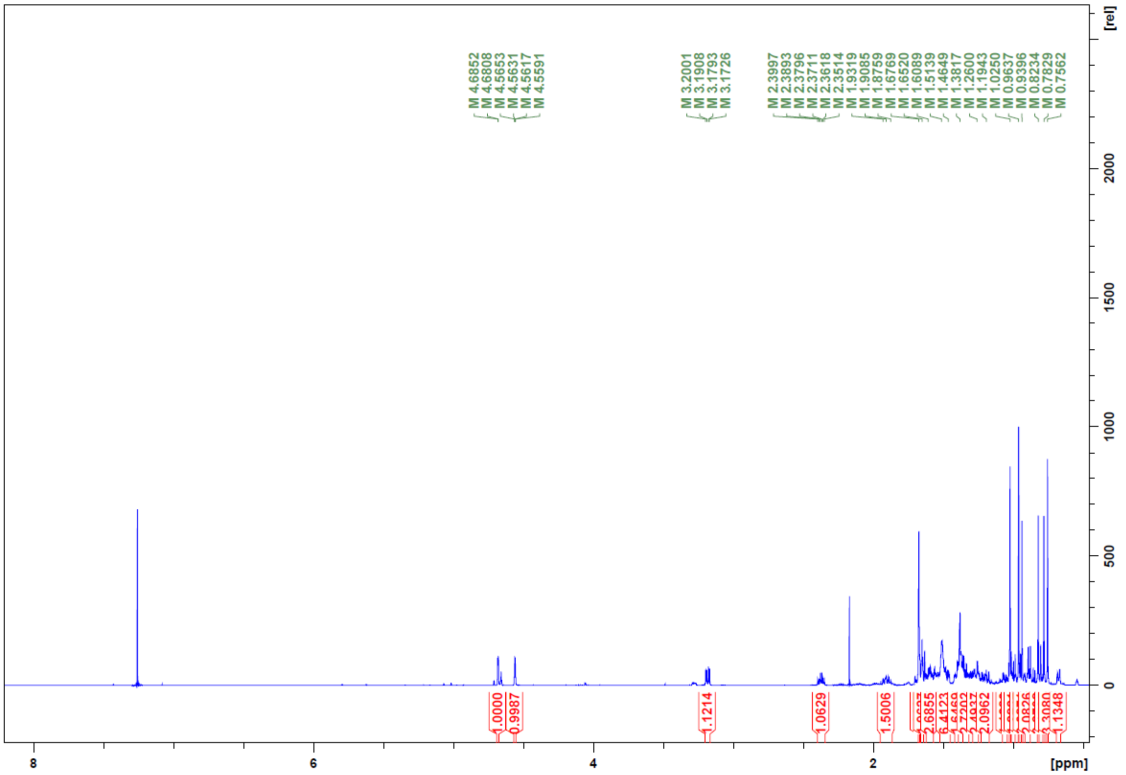

Figure S12. ^{1}H NMR spectrum of **8** in $CDCl_3$.

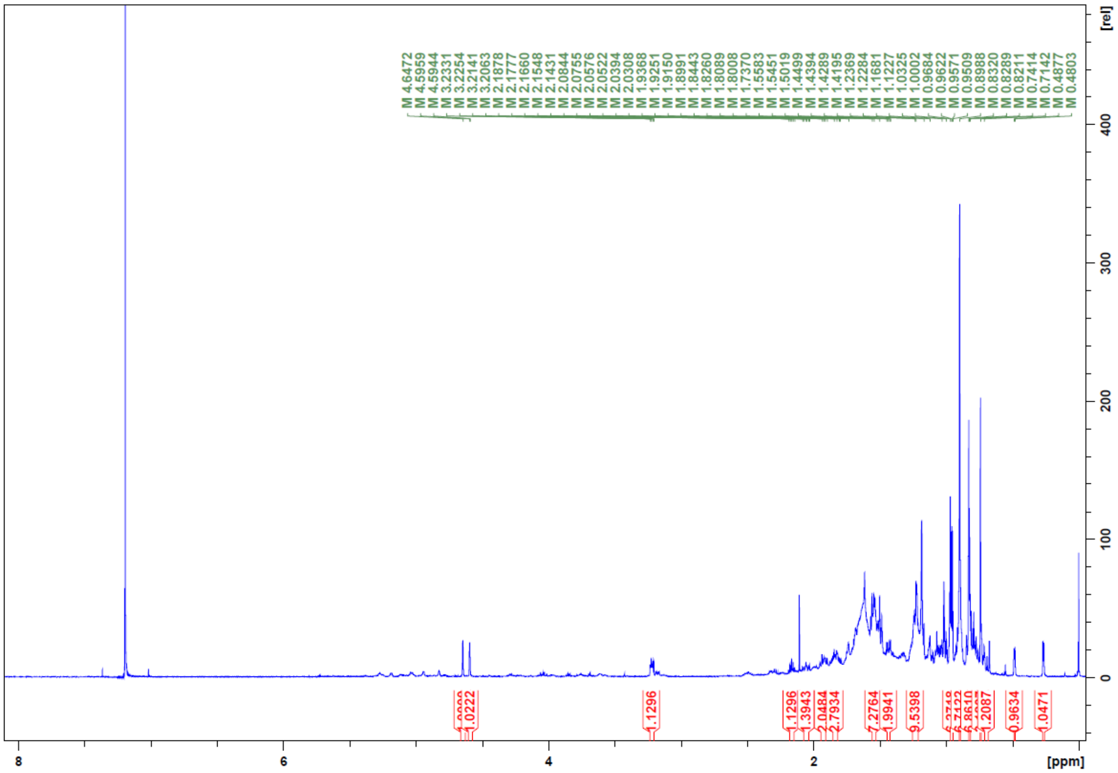

Figure S13. ^{1}H NMR spectrum of **9** in $CDCl_3$.

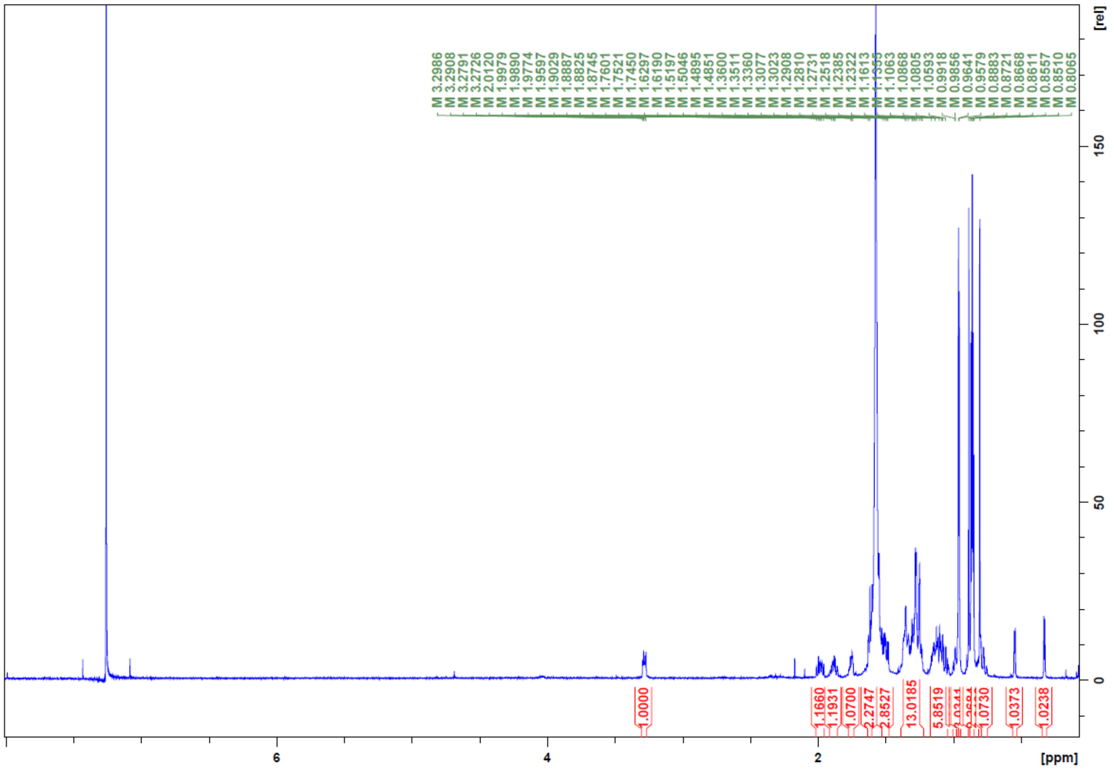

Figure S14. ^{1}H NMR spectrum of **10** in $CDCl_3$.

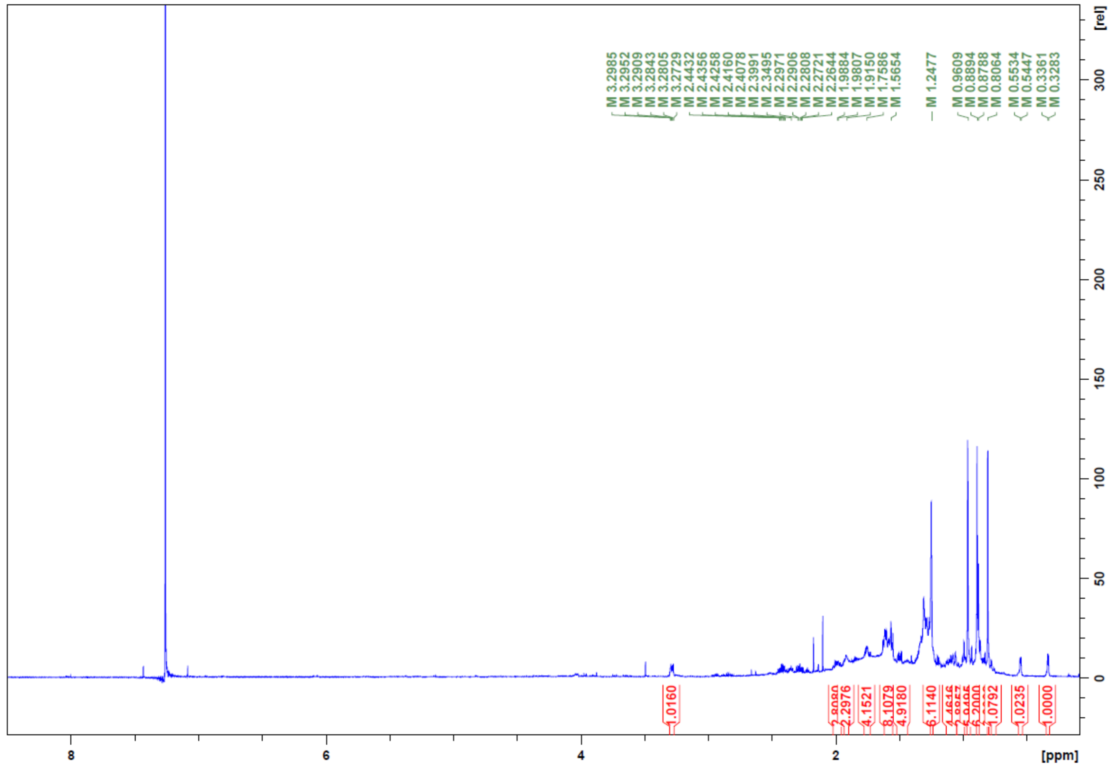

Figure S15. ^{1}H NMR ^{1}H NMR spectrum of a mixture of **11** and **12** in $CDCl_3$.

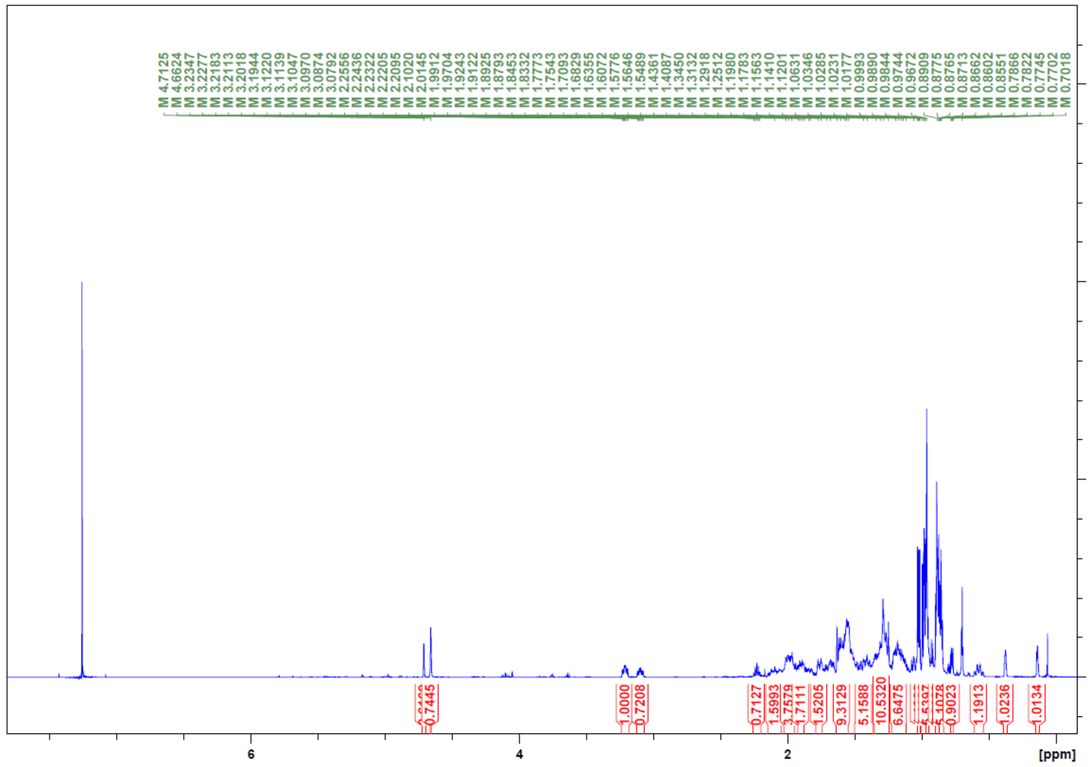

Figure S16. ^{1}H NMR spectrum of a mixture of **13** and **14** (1:0.8) in $CDCl_3$.

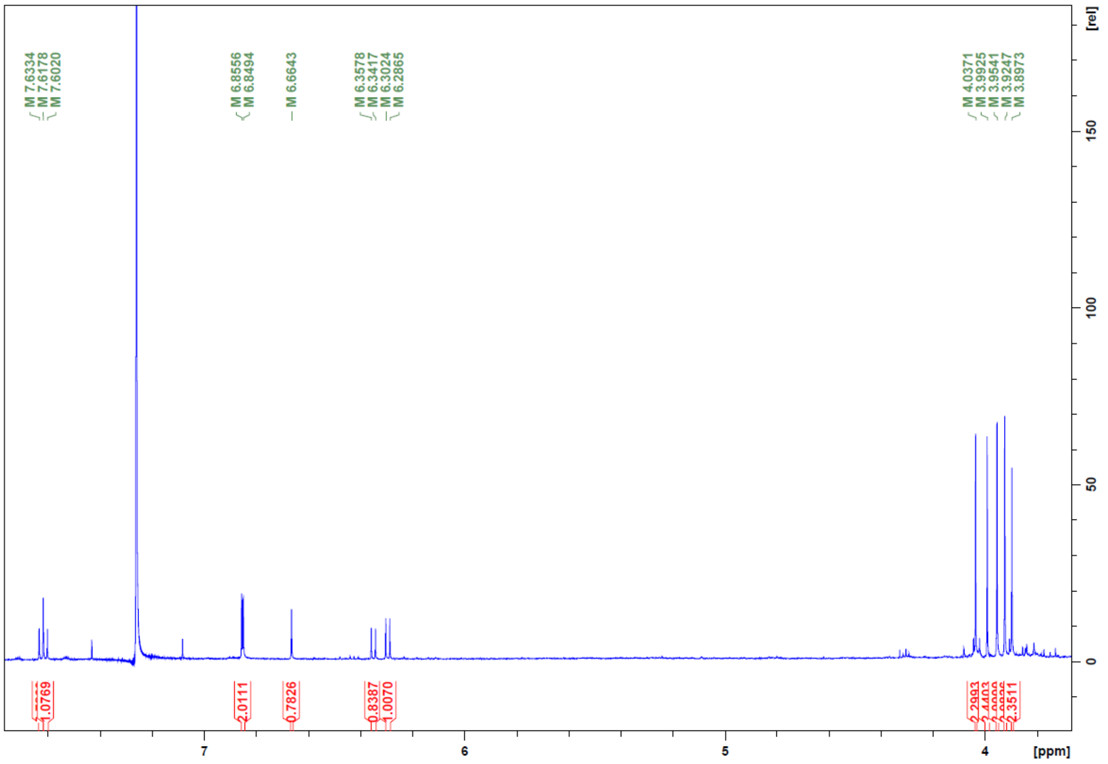

Figure S17. ^{1}H NMR spectrum of **15** in $CDCl_3:CD_3OD$ (1:1).

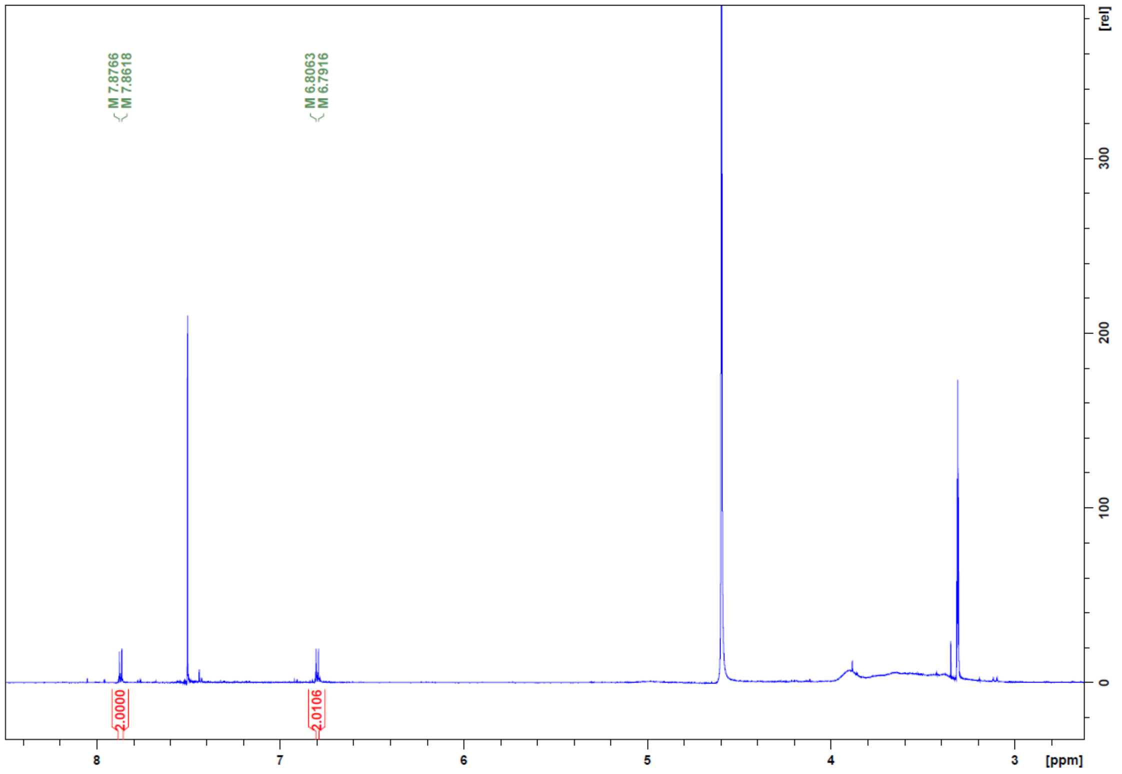

Supplement: Supplementary file 1 [file molecules-27-02206-s001.zip › molecules-1661720-supplementary.pdf]
